# Carbon Dots-Modified Hollow Mesoporous Photonic Crystal Materials for Sensitivity- and Selectivity-Enhanced Sensing of Chloroform Vapor

**DOI:** 10.1007/s40820-024-01598-9

**Published:** 2024-12-26

**Authors:** Junchen Liu, Ji Liu, Zhipeng Li, Liupeng Zhao, Tianshuang Wang, Xu Yan, Fangmeng Liu, Xiaomin Li, Qin Li, Peng Sun, Geyu Lu, Dongyuan Zhao

**Affiliations:** 1 State Laboratory On Integrated Optoelectronics, College of Electronic Science and Engineering, Jilin University, Changchun, 130012 People’s Republic of China; 2 Department of Chemistry and Laboratory of Advanced Materials, Shanghai Key Laboratory of Molecular Catalysis and Innovative Materials, State Key Laboratory of Molecular Engineering of Polymers, Collaborative Innovation Center of Chemistry for Energy Materials (2011-iChEM), College of Chemistry and Materials, Fudan University, Shanghai, People’s Republic of China; 3 School of Engineering and Built Environment, Griffith University, Nathan, QLD 4111 Australia

**Keywords:** Carbon dots, Photonic crystal sensors, Sensitivity-enhanced sensing, Selectivity-enhanced sensing, Chloroform vapor sensing

## Abstract

Chloroform and other volatile organic pollutants have garnered widespread attention from the public and researchers, because of their potential harm to the respiratory system, nervous system, skin, and eyes. However, research on chloroform vapor sensing is still in its early stages, primarily due to the lack of specific recognition motif. Here we report a mesoporous photonic crystal sensor incorporating carbon dots-based nanoreceptor (HMSS@CDs-PCs) for enhanced chloroform sensing. The colloidal PC packed with hollow mesoporous silica spheres provides an interconnected ordered macro-meso-hierarchical porous structure, ideal for rapid gas sensing utilizing the photonic bandgap shift as the readout signal. The as-synthesized CDs with pyridinic-N-oxide functional groups adsorbed in the hollow mesoporous silica spheres are found to not only serve as the chloroform adsorption sites, but also a molecular glue that prevents crack formation in the colloidal PC. The sensitivity of HMSS@CDs-PCs sensor is 0.79 nm ppm^−1^ and an impressively low limit of detection is 3.22 ppm, which are the best reported values in fast-response chloroform vapor sensor without multi-signal assistance. The positive response time is 7.5 s and the negative response time 9 s. Furthermore, relatively stable sensing can be maintained within a relative humidity of 20%–85%RH and temperature of 25–55 °C. This study demonstrates that HMSS@CDs-PCs sensors have practical application potential in indoor and outdoor chloroform vapor detection.

**Supplementary Information:**

The online version contains supplementary material available at 10.1007/s40820-024-01598-9.

## Highlights

- Uniform-sized hollow mesoporous silica spheres form colloidal photonic crystals for gas sensing.
- ‘Nanoreceptors’ have been introduced for increasing the sensing sensitivity and specificity.
- A chloroform gas sensor is created with a sensitivity of 0.79 nm ppm^−1^ with a limit of detection of 3.22 ppm, which are the best reported values in fast-response chloroform vapor sensors without multi-signal assistance.

**Supplementary Information:**

The online version contains supplementary material available at 10.1007/s40820-024-01598-9.

## Graphical abstract

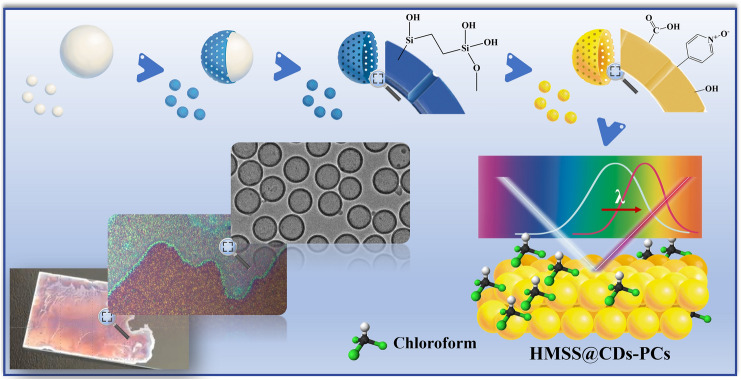

## Introduction

Chloroform is an organic compound widely used as a solvent in factories and laboratories [1], and it is also a precursor for chemical products and pharmaceuticals [2]. Chloroform is volatile and decomposes into highly toxic phosgene when exposed to light [3]. When it enters the human body through the digestive tract, respiratory tract, or skin contact, it affects the central nervous system and can damage the heart, liver, and kidneys. Therefore, real-time monitoring of chloroform gas is crucial [4]. Previous research has shown that chloroform has high chemical inertness. As a result, the use of metal oxide semiconductor (MOS) sensors [5], electrochemical sensors [6], surface plasmon resonance (SPR) sensors [7], and fluorescence sensors [8], which rely on chloroform’s chemical properties for detection, presents significant challenges. These sensors often suffer from disadvantages such as low sensitivity, low selectivity, and high detection limits [9].

Photonic crystals (PC) are micro-/nano-structured materials formed by periodic arrangement of material media with various refractive indices [10]. They possess a photonic band gap (PBG) [11], within which photons of energies in the PBG frequency range cannot propagate through the PC. The waveguiding effect induced by the PBG makes PC systems highly sensitive to environmental changes, as these changes can shift the wavelength of the reflected spectrum. Nature has utilized this light–matter interactions for stimuli responses, such as camouflage, aposematic predator deterrence, territorial assertions, and courtship [12]. This has spurred strong research interest in biomimetic optical sensors [13, 14]. Colloidal photonic crystal (CPC) formed by wet chemistry colloidal self-assembly offers the opportunity of creating hierarchical-ordered porous structures through packing micro- or mesoporous size monodispersed particles or via structural inversion [15, 16]. This unique hierarchically ordered PBG structure is ideal for gas sensing because of the interconnected microporous framework excellent for mass transfer and the micro- or mesopores on the walls that are ideal for trapping analyte gas molecules. When the gas molecules diffuse into the sensor and the analyte gas molecules selectively trapped in the micro- or mesopores on the walls, they alter the average effective refractive index of the system, e.g., chloroform refractive index 1.44 c.f. air 1, causing a shift in the diffraction peak of the PC [17]. Therefore, both qualitative and quantitative detection of gas can be achieved by measuring the shift in the diffraction peak [18].

However, in practical outdoor applications for detecting chloroform vapor, the sensitivity and selectivity of PC sensors are often poor, due to the lack of specific receptors to the analytes. Using conventional chemical functional groups lacks the specificity, while employing protein-based receptors could fall short in stability and cost-effectiveness. In this work, we have introduced nanoparticle-based ‘receptors’ for increasing the sensing sensitivity and specificity. Carbon dots (CDs) are nanomaterials composed of carbon skeletons, typically comprising a mixture of hybrid graphite carbon and amorphous carbon, with particle sizes below 10 nm and a rich, tunable variety of surface functional groups [19–22]. In previous studies, CDs have demonstrated rapid photo-generated electron transfer capabilities, exceptional fluorescence properties, and have been successfully employed as efficient electron acceptors and donors. The functional groups on CDs assume a three-dimensional spatial arrangement, which provides additional spatial confinement for sensing specificity. Importantly, the dimension of CDs matches perfectly with mesopores (2–50 nm); therefore, CDs can be embedded in mesopores, functioning as ‘nanoreceptors.’

Another challenge in wet chemistry-based fabrications is to form large-area, uniform colloidal PC films [23]. During the self-assembly process of colloidal particles to form PC films, cracks are often prevalent. These cracks and other structural defects significantly impact the performance of the sensors. Though these defects may play a role of waveguides in some cases, the randomness of crack appearance significantly affects the consistency of sensing signals, which requires new strategies for circumventing crack formation [24]. Additionally, hierarchically ordered CPC sensors have the flexibility in constituent particle morphology and chemistry design that can accommodate different functional group modifications, providing unique advantages in gas detection selectivity [25, 26].

This study presents the microscopic design of the structural unit of PC gas sensors. The sensor features a CPC comprised of hollow mesoporous silica spheres (HMSS) modified by CDs with pyridine N-oxide groups on surface, which have a specific affinity for chloroform vapor [27]. Moreover, the CDs were found to function as a molecular glue that significantly reduced film cracking during the self-assembly process. As a result, a PC gas sensor with a high yield, high sensitivity, high selectivity, and low detection limit for chloroform vapor (HMSS@CDs-PCs) was developed. More specifically, uniform mesoporous silica nanoparticles were prepared using the soft templating method. Then, hollow structures were fabricated using the hard templating method. CDs were synthesized by carefully selecting appropriate precursors. These CDs had active functional groups, such as carboxyl (-COOH) [28], amino (-NH_2_) [29], hydroxyl (-OH) [30], and pyridine N-oxide groups on their surfaces [27]. These functionalized CDs were then loaded onto the surface of hollow mesoporous microspheres. By utilizing the molecular adhesive properties of the CDs, the self-assembly forces between structural units were enhanced. This minimized cracking during the assembly process, leading to the formation of more perfect PC [31–34].

The real-time optical response to chloroform vapor was measured using a reflectance spectrum test platform equipped with an optical fiber spectrometer, showing superior sensitivity and selectivity of HMSS@CDs-PCs toward chloroform. Modifying the HMSS with CDs exhibiting pyridinic-N-oxide groups on surface not only affords selectivity to chloroform, but also enabled the formation of large-area, crack-free PC films. The study illustrates the versatility of hollow mesoporous silica spheres as a gas sensor platform, as well as demonstrates the extraordinary multi-functionalities of CDs in sensor device fabrications. The approach described here holds promise for real-world applications in environmental monitoring, biomedical sciences, and industrial safety.

## Experimental Section

### Materials

Ethyl alcohol (EtOH, AR), concentrated ammonia solution (28 wt%), tetraethyl orthosilicate (TEOS), cetyltrimethylammonium bromide (CTAB, ≥ 99%), 1,2-bis(triethoxysilyl)-ethane (BTEE, 96%), sodium hydroxide (NaOH, AR), anhydrous citric acid (AR), and dicyandiamide (≥ 99%) are analytical-grade reagents purchased from Shanghai Aladdin Biochemical Technology Co., Ltd. All other chemicals were used as received.

### Preparation of SiO_2_, HMSS, CDs, and HMSS@CDs

#### ***Preparation of Silica (SiO***_***2***_***)***

SiO_2_ nanospheres were synthesized using a modified version of Stöber method [35]. In a 500-mL round-bottomed flask, 100 mL of EtOH, 10 mL of deionized water, and 8 mL of concentrated ammonia solution were mixed. The solution was stirred for 10 min. Subsequently, a mixture of 7.5 mL of TEOS and 25 mL of EtOH was quickly added to the flask. The solution was stirred for 6 h at a rate of 650 rpm. Then, the white product obtained was washed with EtOH and centrifuged three times. The resulting white product was dispersed in 30 mL of EtOH and designated as SiO_2_-aq.

#### Preparation of Hollow Mesoporous Silica Spheres (HMSS)

HMSS were synthesized using the template method [36]. Firstly, CTAB micelles were formed in solution. In a round-bottomed flask with a volume of 250 mL, 30 mL of deionized water, 0.06 g of CTAB, and 0.4 mL of concentrated ammonia solution were mixed. The solution in an oil bath at 35 °C was stirred for 15 min. Secondly, CTAB micelles were uniformly assembled on the surface of the inorganic silica nanoparticles. A mixture of 1 mL of SiO_2_-aq and 11 mL of EtOH was slowly added to the flask. The solution was stirred for 30 min at a rate of 600 rpm. Thirdly, a smooth and uniform organic mesoporous silica layer was formed. After 30 min of stirring, 0.1 mL of BTEE was slowly added to the flask. After 4 h of stirring, the resulting white product was washed with ethanol and centrifuged three times. The white product was dispersed in 20 mL of deionized water and named MSS-aq. Finally, the hollow structure is obtained by selective etching of the internal inorganic silica. Subsequently, 0.38 g of NaOH was added to MSS-aq and stirred for 40 min at room temperature. The resulting product was washed with deionized water and centrifuged twice. The white product was dispersed in 10 mL of deionized water and named HMSS-aq.

#### Preparation of CDs

N-doped CDs were synthesized using a hydrothermal method. 1 g of anhydrous citric acid (CA) and 1 g of dicyandiamide were dissolved in 5 mL of ultrapure water. The solution was then transferred into a 25-mL polytetrafluoroethylene vessel in a hydrothermal reactor. The reactor was placed in an oven at 180 °C for 12 h. After cooling to room temperature, the solution was transferred to a dialysis bag with a molecular weight cutoff of 3500 Da and dialyzed for 48 h. During dialysis, the ultrapure water outside the dialysis bag was constantly replaced. After dialysis, the CD solution, labeled CDs-aq, was collected and stored in a refrigerator at 4 °C.

#### Preparation of HMSS@CDs

The mixture consisting of 10 mL HMSS-aq and 10 mL CDs-aq was placed in an ultrasonic cleaning machine with an ultrasonic power of 150 W and an ultrasonic frequency of 40 kHz and was ultrasonicated for 30 min. The resulting light-yellow product was washed once with ethanol. The product was dispersed in 10 mL of ethanol and labeled HMSS@CDs-aq.

### Characterization

The crystal structures of the prepared samples were analyzed using powder X-ray diffraction (XRD, Rigaku D/MAX-2550, Cu Kα, λ = 1.5418 Å). The surface morphology of the samples was characterized by field emission scanning electron microscopy (SEM, JEOL JSM-7500F). Transmission electron microscopy (TEM) was conducted on a JEOL JSM-2100F microscope to determine the morphology of as-prepared samples. X-ray photoelectron spectroscopy (XPS) measurements were performed in a VG ESCALAB 210 (VG Scientific) photoelectron spectrometer equipped with a Mg K source. The instrument used for specific surface area and pore size analysis (BET) is QUADRASORB evo, a fully automatic specific surface area and pore analyzer from Quantachrome Company, USA. The total specific surface area was calculated by the BET method, and the pore diameter was calculated by the BJH method. A Nicolet IS50 Fourier transform infrared spectrometer (FT-IR) (Thermo Fisher Scientific, USA) was used to analyze the surface functional groups of samples. The freeze-dried CDs powder (CDs-s) and potassium bromide powder were ground together, and then, the mixture was made into transparent flakes and analyzed in an FI-IR spectrometer.

### Fabrication and Measurement of the Sensors

The reflection spectroscopy system was purchased from Shanghai Simtrum Technology Co., Ltd. All high-purity (99%) test gases were obtained from Shanghai Wetry Standard Gas Analysis Technology Co., Ltd. A volume of 0.2 mL of HMSS@CDs-aq was spread onto a plasma-treated quartz sample cell. After drying at room temperature, a PC film, named HMSS@CDs-PCs, formed in the quartz sample cell. HMSS@CDs-PCs and the reflectance spectroscopy system were packaged in a homemade sealed chamber (Fig. S1). The sensitivity of a PC gas sensor can be defined as the amount of change in the reflected spectrum when the measured gas changes one unit concentration. The sensitivities of the four PC sensors (SiO_2_-PCs, MSS-PCs, HMSS-PCs, and HMSS@CDs-PCs) were compared in a closed container filled with chloroform vapors. The sensitivity calculation is shown in below:

1 $$\begin{array}{*{20}c} {S_{n} = \frac{\Delta \lambda }{{\Delta C}}} \\ \end{array}$$

where $S_{n}$ is the sensitivity of PC sensor, $\Delta C$ is the change in the concentration of the detected gas, and $\Delta \lambda$ is the change in the reflection peak of the sensor when the concentration of the detected gas changes. It represents the shift in the reflection peak when the concentration of the detected gas changes by one unit.

The limit of detection (LOD) is defined as the lowest concentration of the target gas component that can be detected in a sample. It corresponds to the gas concentration that produces a redshift in the reflected light signal equal to three times the standard deviation of the baseline noise (Fig. S2), which can be expressed as:

2 $$\begin{array}{*{20}c} {{\text{LOD}} = \frac{3\sigma }{{S_{n} }}} \\ \end{array}$$

where $\sigma$ is calculated the noise value, which is 0.85 nm. Under the same concentration but different types of gas atmospheres, the difference in sensitivity of the PC sensor can be used to evaluate the selectivity of the sensor. The larger the sensitivity difference, the better the selectivity. Response time refers to the time it takes for the detector to reach 90% of the stable indicated value after contacting the measured gas. Recovery time refers to the time it takes for the detector to return to 10% of the stable indicated value after being separated from the measured gas.

## Results and Discussion

### Manufacture of Sensitivity- and Selectivity-Enhanced PC Gas Sensor

**Fig. 1:**
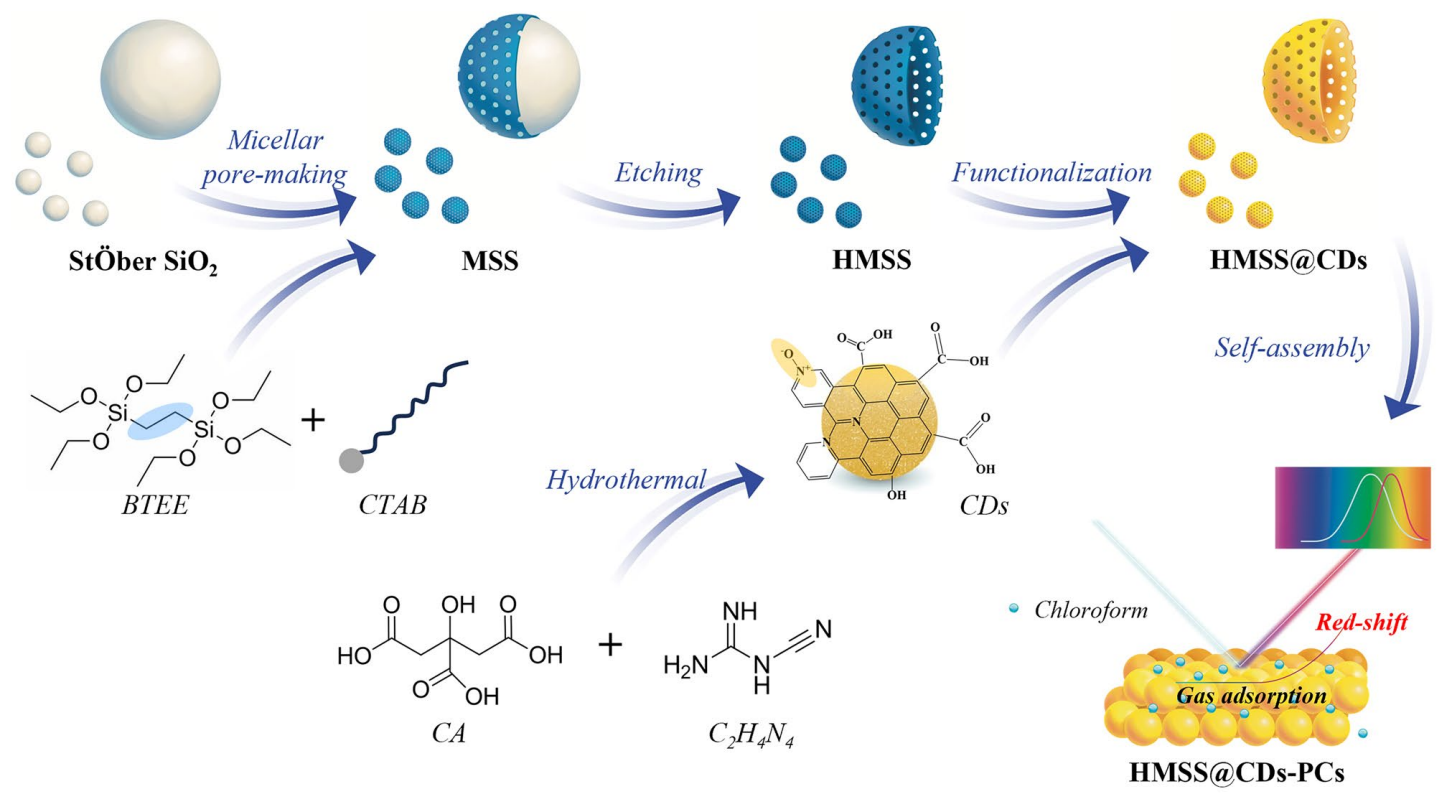
Schematic of the preparation procedures for the hollow mesoporous PC sensing film (HMSS@CDs-PCs). Monodisperse inorganic silica microspheres prepared by a modified Stöber method were used as hard templates for the hollow structure, while CTAB micelles served as the soft template for the mesoporous structure. Ethylenediamine provided the nitrogen source to prepare CDs containing a pyridine N-oxide functional group

In the synthesis, the organic silica shell over the inorganic silica core is key to forming hollow mesoporous silica. After synthesizing the silica core particles, CTAB was introduced into the solution to form micelles. These micelles attached to the surface of the inorganic silica spheres, serving as soft templates for mesopores [37]. The organic silane BTEE undergoes hydrolysis, using the micelle-coated inorganic silica spheres as cores to form organic silica shell layers. Subsequently, ethanol and water were used to wash away the CTAB micelles, creating mesoporous structures in the organic silica shell layer. Due to the different alkali resistances of organic and inorganic silica, the inorganic silica core was then etched away with 0.5 M NaOH, forming a hollow structure. The exposed functional groups of the organic silica adsorb CDs and form hydrogen bonds, resulting in composite materials (HMSS@CDs). These HMSS@CDs particles were then arranged into a PC sensor (HMSS@CDs-PCs) through self-assembly (Fig. 1).

**Fig. 2:**
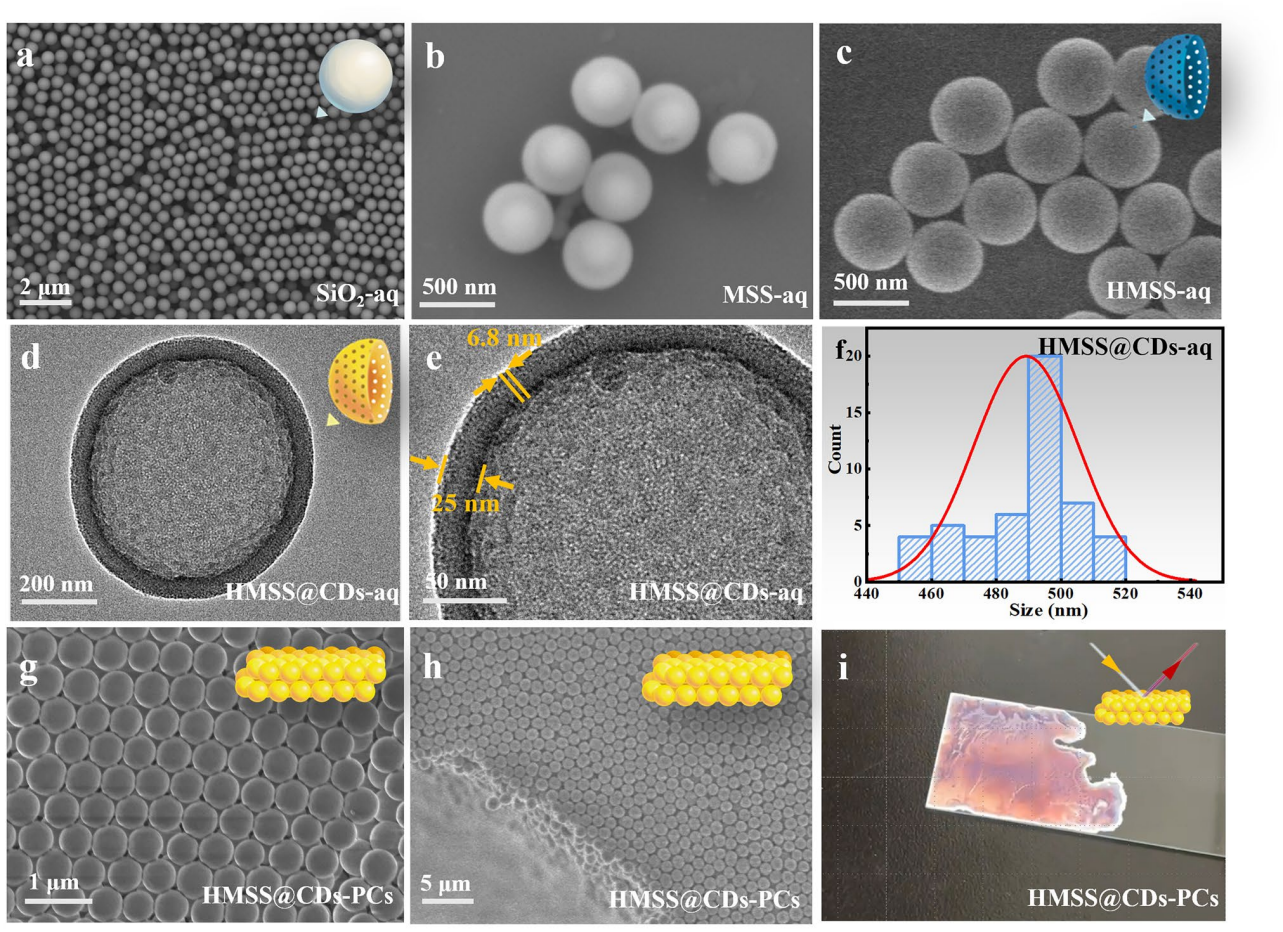
Morphology characterization. **a** SEM image of SiO_2_-aq, i.e., the inorganic silica spheres of 470 nm diameter that has not undergone calcination with abundant silanol groups on surface. **b** SEM image of MSS-aq, i.e., CTAB micelles serve as a soft template, allowing the organosilica shell to form a mesoporous structure during the encapsulation of inorganic silica. The MSS-aq exhibits a distinct core–shell structure with a particle size of approximately 500 nm. **c** SEM image of HMSS-aq formed by selective alkali etching utilizing the greater etching resistance of organosilia compared to inorganic silica. **d** TEM image of HMSS@CDs-aq. The modification with CDs does not damage the original structure. **e** a close-up TEM image of HMSS@CDs-aq showing discernible mesoporous pores. Inset shows the pore size distribution. **f** Particle size distribution of HMSS@CDs-aq. The particles exhibit good uniformity, 490 nm. **g, h** SEM images of HMSS@CDs-PCs. The particles are arranged in face-centered cubic order, both on the surface and in cross section. **i** Optical photograph of the HMSS@CDs-PCs film for a more straightforward description

The monodisperse SiO_2_-aq microspheres, with a sphere diameter of approximately 470 nm (Fig. 2a), were prepared using an improved Stöber method. The SEM image shows the distinct core–shell structure of MSS-aq (Fig. 2b). The darker outer shell consists of organosilica, while the brighter inner core consists of inorganic silica. The average sphere diameter of MSS is approximately 500 nm. NaOH was used to etch away the SiO_2_, taking advantage of the higher alkali resistance of organosilica. The organosilica shells remained intact, preserving their spherical structures.

The sphere diameter remains around 500 nm, unchanged compared to before etching (Fig. 2c). After the functionalization of CDs, the core–shell structure remains intact, with a particle size of approximately 500 nm (Fig. 2d). After NaOH etching, the silica shell will shrink slightly, resulting in a slight reduction in the final size. The shell thickness is about 25 nm (Fig. 2e).

CTAB is a cationic surfactant that acts as a templating agent in the synthesis of mesoporous silica nanoparticles. The structure of CTAB contains a hydrophilic head group and a hydrophobic tail, allowing it to form micelle structures in solution. Under alkaline conditions, CTAB micelles interact with the silica source BTEE through electrostatic and hydrophobic interactions to form complexes. This complex formation specifically enables the silica source to organize into an ordered, vertically aligned mesoporous structure under the guidance of the CTAB template [36]. The pore size of the mesopores is approximately 6.8 nm (Fig. 2e).

The sphere diameter distribution analysis shows that the hollow HMSS@CDs-aq spheres have excellent size consistency, with a uniform diameter of approximately 500 nm (Fig. 2f). The monodisperse HMSS@CDs-aq particles were dispersed in ethanol and then dripped onto a quartz glass substrate, where they self-assembled into a PC film as the ethanol evaporated. The microstructure of the self-assembled HMSS@CDs-PC indicated a good self-assembly base on a face-centered cubic (FCC) order of particles arranged (Fig. 2g) and an FCC order of cross section (Fig. 2h). Due to the well-ordered periodic arrangement, the optical photograph of the HMSS@CDs-PC film exhibits excellent PC effects, reflecting brilliant colors (Fig. 2i). The film’s color changes with the angle of incident light, further proving good self-assembly (Video S1).

Due to the aggregation-caused quenching (ACQ) effect of CDs, the luminescence of CDs weakens or even disappears completely in concentrated solutions or an aggregated state. A comparison was made between the fluorescence effects of HMSS@CDs-aq in ethanol solution and after forming a HMSS@CDs-PC film (Fig. S3). Under 365 nm ultraviolet light irradiation, fluorescence is observed in HMSS@CDs-PCs. This shows that CDs are dispersedly distributed in HMSS. The nanoscale channels and void structures provided by HMSS allow the CDs to disperse within these voids, forming a stable dispersed state. The ACQ effect caused by CD accumulation can be alleviated, so as to maintain fluorescence [38].

**Fig. 3:**
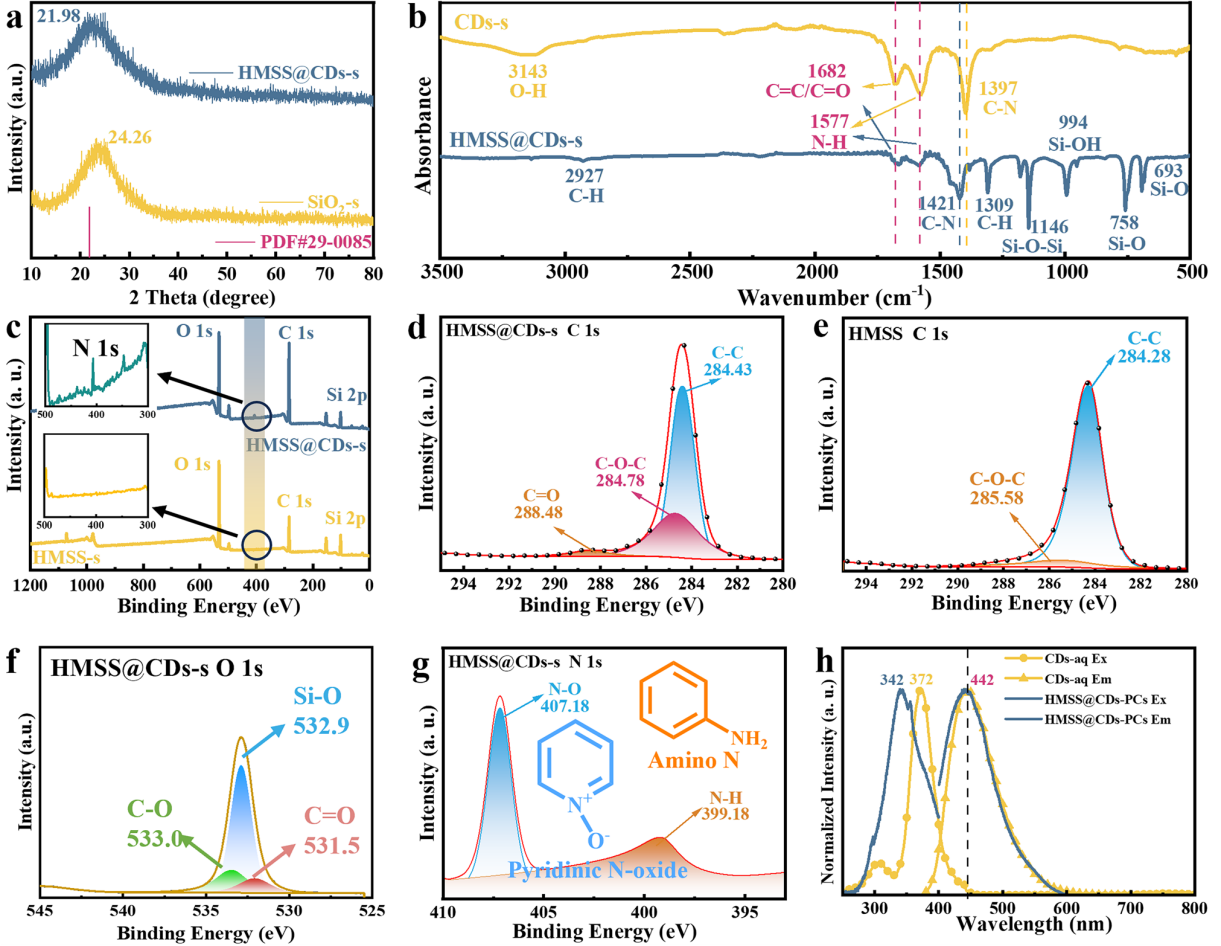
Structural characterization. **a** XRD patterns of the CDs-functionalized hollow mesoporous silica sphere powder (HMSS@CDs-s) after drying treatment, compared with Stöber SiO_2_ powder (SiO_2_-s) and the standard amorphous silica PDF card (PDF#29–0085). **b** FI-IR spectra of HMSS@CDs-s and CDs-s pelleted with potassium bromide (KBr). **c** XPS survey spectra of HMSS@CDs-s and HMSS-s. The significant increase in the carbon content of HMSS@CDs-s reflects the surface modification of the CDs. **d** XPS high-resolution C 1*s* spectrum of HMSS@CDs-s. The peak at 288.48 eV corresponding to C = O bond which is characteristic peak of CDs. **e** XPS high-resolution C 1*s* spectrum of HMSS-s. **f** XPS high-resolution O 1*s* spectrum of HMSS@CDs-s. **g** XPS high-resolution N 1*s* spectrum of HMSS@CDs-s. The strong peak at 407.18 eV corresponds to pyridinic N-oxide, while a small peak at 399.81 eV can be attributed to amino. **h** PL spectra of CDs-aq and HMSS@CDs-PCs

The wide-angle XRD spectra of HMSS@CDs-s (Fig. 3a) matched well with the amorphous silica (PDF#29–0085). The 2θ of the (111) peak located at 24.26° for SiO_2_-s shifted markedly toward higher angles compared with the original 21.98°. This peak shift can be attributed to the presence of residual stress in the material. Compared with SiO_2_-s, the intensity of the peak corresponding to the Si–OH of HMSS@CDs-s is weakened. This is due to two main factors: first, the shell of HMSS@CDs-s is composed of organic silica in which two Si atoms are linked by -C = C-, reducing the number of Si–OH groups [39]. On the other hand, the core composed of inorganic silica is etched away in NaOH etching, further reducing the number of Si–OH [40]. The pore size of the mesoporous shell is related to the CTAB hydrolysis time and hydrolysis temperature [41]. As can be seen from the small-angle XRD patterns of HMSS-s and HMSS@CDs-s (Fig. S12), there are obviously sharp diffraction peaks at 2θ of 1.2°. This diffraction peak is the (110) plane diffraction peak of two-dimensional hexagonal mesoscopic structure, which is consistent with the diffraction peak of mesoporous material FDU 15–1400 [42]. The diffraction peak at 7.4° can be attributed to (200) diffraction of two-dimensional hexagonal mesoscopic structure. The results show that the mesoporous structures of HMSS-s and HMSS@CDs-s are ordered. As observed from the nitrogen adsorption–desorption isotherms (Fig. S11), TEM images, and small-angle XRD patterns, the pore size of HMSS@CDs is approximately 6–10 nm.

The FI-IR spectra of CDs-s and HMSS@CDs-s were obtained (Fig. 3b). The characteristic absorption bands of CDs-s at 3143 and 1397 cm^−1^ correspond to the stretching vibrations of O–H groups and -C-N groups. The characteristic absorption bands of HMSS@CDs-s at 1146 and 994 cm^−1^ are attributed to the stretching vibrations of Si–O-Si and Si–OH, while peaks at 758 and 693 cm^−1^ correspond to the stretching vibrations of -Si–O. The peaks at 2927 and 1309 cm^−1^ correspond to the stretching vibrations of -C-H. Comparing the infrared spectra of these two materials, the characteristic absorption bands of CDs-s and HMSS@CDs-s at 1682 and 1577 cm^−1^ correspond to the stretching vibrations of the -C = C/-C = O and N–H groups, indicating that abundant -NH_2_ and -COOH groups were present on their surface. In particular, the -C-N stretching vibration peak of HMSSCDs-s is located at 1421 cm^−1^, which is significantly blue-shifted compared to 1397 cm^−1^ of CDs-s. This is because CDs are grafted on the surface of HMSS, and the coplanarity of the conjugated system between CD molecules is destroyed [43]. The steric hindrance limits the conjugation effect, causing the absorption frequency to shift to higher wavenumbers. FT-IR spectroscopy analysis indicates that the surface of HMSS@CDs-s contains functional groups such as silanol group and pyridine N-oxide group.

The chemical composition of HMSS@CDs-s was investigated by XPS. The XPS survey spectra (Fig. 3c) clearly show the difference in the type and content of elements of the HMSS@CDs-s (after CDs modification) and the HMSS (before CDs modification), while the survey spectra of HMSS@CDs-s are comprised of silicon (12.35%), carbon (58.27%), oxygen (27.54%), and nitrogen (1.85%). The survey spectra of HMSS-s consist of silicon (22.82%), carbon (34.63%), and oxygen (42.39%). The significant increase in the carbon content of HMSS@CDs-s reflects the surface modification of the CDs. To visually compare the modification of the material by the CDs, the XPS spectra of C 1*s* and O 1*s* for HMSS@CDs and HMSS were compared. The C 1*s* spectrum of HMSS@CDs-s can be fitted with three peaks: the strongest peak at 284.43 eV attributed to C–C bond, the peak at 284.78 eV ascribed to C–O–C bond, and a third (weakest) peak at 288.48 eV corresponding to C = O bond (Fig. 3d). The C 1*s* spectrum of HMSS can be fitted with two peaks: a strong peak at 284.28 eV attributed to C–C bond and a weak peak at 285.58 attributed to the C–O–C bond (Fig. 3e). The high-resolution O 1*s* spectrum of HMSS@CDs-s can be deconvoluted into three peaks (Fig. 3f): Si–O (532.90 eV), organic C = O (531.50 eV) and organic C–O (533.0 eV). In contrast, the high-resolution O 1*s* spectrum of HMSS-s can be deconvoluted into two peaks (Fig. S6): Si–O (532.80 eV) and organic C–O (533.0 eV). The characteristic peak of the CDs is the organic C = O. These differences also reflect the surface modification of the CDs. The high-resolution N 1*s* spectrum of HMSS@CDs-s can be fitted into two peaks: the strong peak at 407.18 eV corresponds to pyridinic N-oxide, while a small peak at 399.81 eV can be attributed to an amino group (Fig. 3g). This is because the synthesis process of CDs is in a closed system with high temperature and pressure. A considerable amount of pyridinic nitrogen oxide moiety is produced on the surface of CDs.

Mesoporous materials provide nanoscale channels and pore structures (2–50 nm), allowing CDs to disperse within the pores and form a stable dispersed state, thus preventing excessive aggregation. The photoluminescence (PL) performance of HMSS@CDs-s powder was tested by fluorescence spectrophotometer (Fig. 3h). After CDs are loaded onto the HMSS CPC, the PL effect still exists. The maximum excitation wavelength of HMSS@CDs-s is 342 nm, and the maximum emission wavelength is 442 nm. Compared with CDs-aq dispersed in deionized water, the emission peak position of HMSS@CDs-s did not change significantly, and the excitation peak was blue-shifted by 30 nm. This may be due to two aspects. On the one hand, there is a certain degree of aggregation of CDs, resulting in a blueshift of the excitation wavelength. When CDs aggregate, the interactions among multiple particles lead to a decrease in the spatial confinement size. The reduction in spatial confinement size enhances the quantum confinement effect of the CDs, which could result in the formation of higher energy states. The enhancement of the quantum confinement effect in CDs increases the energy required for electronic transitions, thereby causing a blueshift in the excitation wavelength [44]. On the other hand, the silane groups possess strong electron-accepting abilities, effectively capturing local electrons near the edges of the CDs [45]. This attraction leads to the aggregation of electrons on the surface or edges of the CDs, forming regions of high electron density. As electrons accumulate at the edges of the CDs, the energy states of these localized electrons change. This alteration results in an increase in the energy of the conduction band, raising the energy required for transitions from the valence band to the conduction band [46]. With the elevation of the conduction band energy, the energy required for electronic transitions increases, thereby affecting the absorption and emission characteristics of light. As the conduction band energy rises, the energy of the excited states increases, leading to a shift of the emitted wavelength toward shorter wavelengths, which causes a blueshift in the excitation peak.

**Fig. 4:**
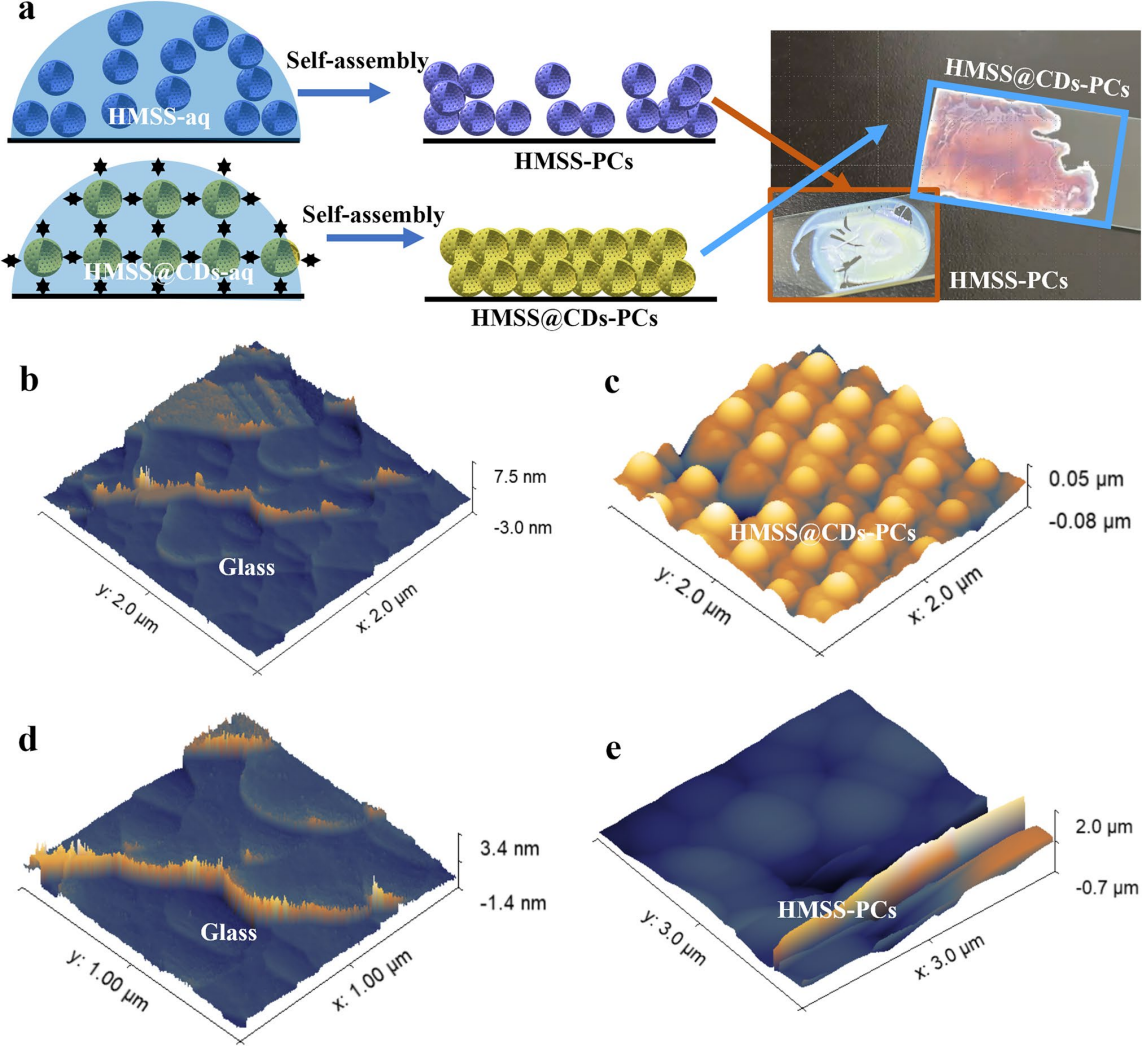
Self-assembly characterization. **a** Schematic and optical photograph of CD as a molecular glue for self-assembly. Ethanol droplets encapsulating monodisperse material particles are applied onto a plasma-treated quartz glass substrate. As the ethanol evaporates at room temperature, the monodisperse particles form a film through self-assembly forces. The CDs provide additional hydrogen bonds, enhancing the self-assembly forces. On the right, the HMSS@CDs-PCs film is smoother, more uniform, and exhibits brilliant structural colors. **b-e** AFM images of glass, HMSS@CDs-PCs, and HMSS-PCs. The maximum roughness of the clean quartz glass substrate is approximately 10 nm, and the maximum roughness of the HMSS@CDs-PCs film on this substrate is about 130 nm

The self-assembly of PC films highly depends on the conventional heterogeneous systems evaporative aggregation. Due to the asymmetry in the drying process, film defects (such as fractures [47], cratering [48], or “coffee rings” [49]) can easily be caused during the preparation of PC thin films. PC films exhibit tunable optical properties. Defects in the films disrupt the periodicity of the ideal PCs, reducing the interference of the periodic structure on light, which leads to scattering and a decrease in color saturation. The types of defects can be classified into point defects, line or planar defects, and surface defects. The defects in the films were observed using an optical microscope, as shown in Fig. S8. Point defects are localized disruptions, such as missing particles or voids (Fig. S8a) [50]. Although point defects are relatively small and may not significantly affect the overall color of the film, they can still produce noticeable color changes if concentrated at critical wavelengths. Point defects typically do not lead to substantial reductions in resolution, but they can cause localized increases in light scattering, resulting in a blur of image details. Line defects or planar defects involve irregular arrangements along a row or layer of the crystal (Fig. S8a) [51]. Line defects can cause large areas of uneven optical response, thereby affecting the interference effects of light. Because they influence the relative positions between multiple layers or particles, they may result in significant changes in the direction and intensity of reflected light, leading to desaturation of color [52]. The presence of line defects alters the propagation path of light, causing variations in the wavelength of the reflected light. Surface defects include cracks, scratches, and particle attachments on the film’s surface (Fig. S8b). As surface defects directly affect incident light in the line of sight, their impact on saturation is usually more pronounced than that of point and line defects [53]. By utilizing CDs to enhance intermolecular forces, a strong covalent coupling is formed between CDs and HMSS molecules (Fig. 4a). Through hydrogen bond-driven assembly into films, it achieves more uniform, brighter, and more saturated structural colors. Minor variations in color saturation are observed across different regions of the HMSS@CDs-PC. Since changes in the reflection peak ($\Delta \lambda$) are used as the sensing signal in this study, initial variations in curve width and peak position are acceptable. When self-assembled on a glass substrate with a maximum roughness of 10.5 nm, HMSS@CDs-PCs demonstrated a maximum roughness of 130 nm. In contrast, the control group HMSS-PCs without CDs as molecular glue had a maximum roughness exceeding 2.7 µm (Fig. 4b, e). This demonstrates that CDs, acting as molecular glue, can enhance the self-assembly forces among the base units.

### Chloroform Sensing Characteristics of PC Gas Sensor

**Fig. 5:**
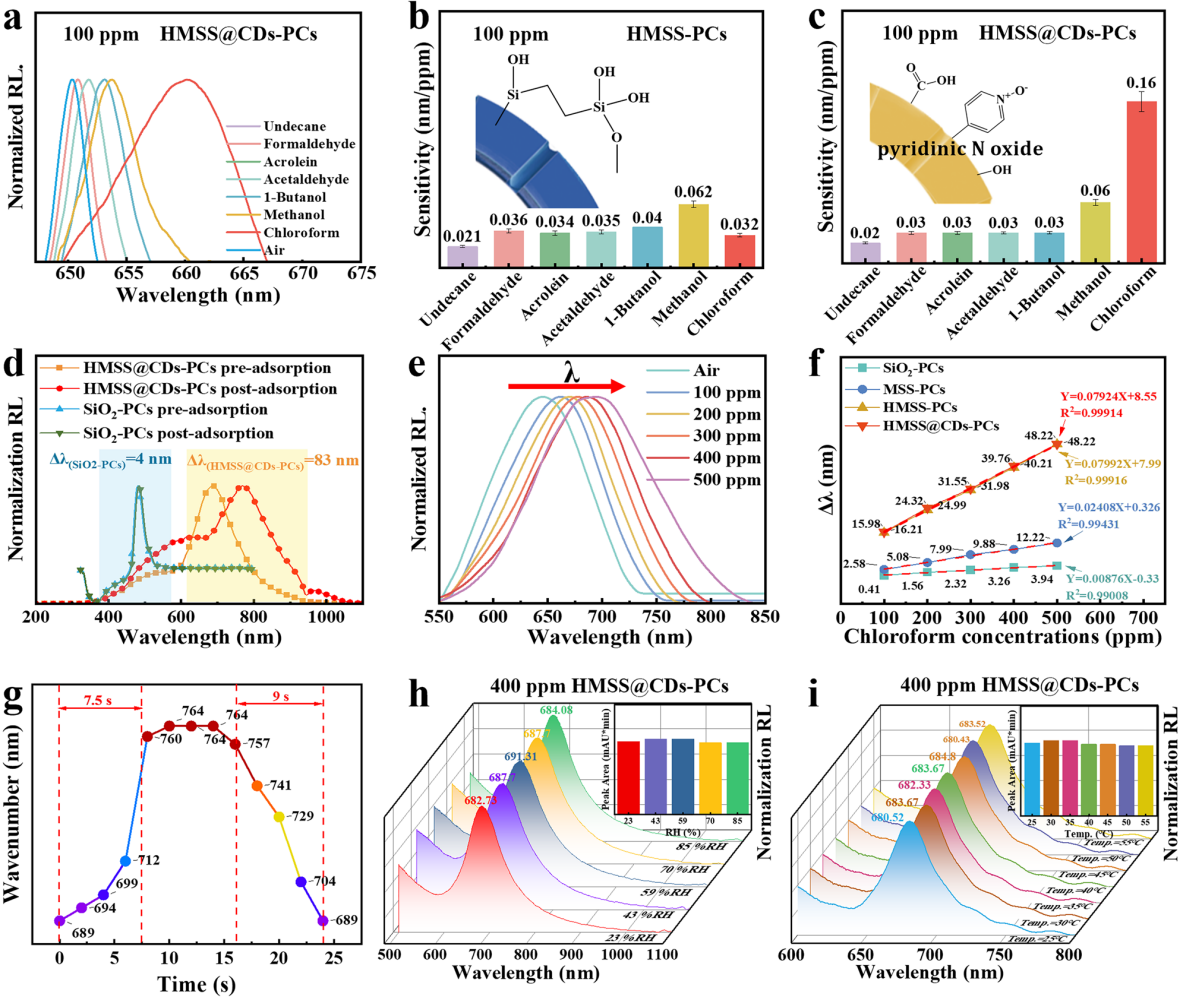
Sensing capability test. **a** Reflection spectrum of HMSS@CDs-PCs sensor under different kinds of 100 ppm VOC atmospheres. **b** Selectivity of HMSS-PCs sensor. **c** Selectivity of HMSS@CDs-PCs sensor. **d** Shift in the reflection peak for HMSS@CDs-PCs sensor and SiO_2_-PCs sensor under 1000 ppm chloroform vapor atmosphere. The reflection peak of SiO_2_-PCs shifted by 4 nm, while that of HMSS@CD-PCs shifted by 83 nm. **e** Reflection wavelength of HMSS@CDs-PCs sensors under different concentrations of chloroform vapor. **f** Sensitivity of PC sensors. The change in the reflection peak of SiO_2_-PCs, MSS-PCs, HMSS@-PCs and HMSS@CDs-PCs sensors under different concentrations of chloroform vapor. The slope of the curve represents the sensitivity of the sensor. **g** Response/recovery speed of HMSS@CDs-PCs sensor. **h** Humidity resistance of HMSS@CDs-PCs sensors under 400 ppm chloroform vapor. **i** Temperature stability of HMSS@CD-PCs sensors under 400 ppm chloroform vapor

To verify the sensing characteristics in complex environments, six VOCs with various functional groups were chosen to test the selectivity of the HMSS@CDs-PCs sensor. In addition to the chloroform vapor, this study also included two alcohols (methanol and 1-butanol), three aldehydes (formaldehyde, acetaldehyde, and acrolein), and an alkane (undecane). These VOCs are often mixed with chloroform in the environment. Acetic acid vapor is a representative acidic gas, while triethylamine is a representative basic gas. The reflection spectra of HMSS@CDs-PCs exhibit varying degrees of redshift under different types of 100 ppm or 500 ppm VOC environments, with the redshift for chloroform being the most pronounced (Figs. 5a, S7, S9). The redshift in the reflection spectrum is primarily due to an increase in the effective refractive index of the PC system [10]. Gases can adsorb onto the film surface and within its pores through van der Waals forces, altering the effective refractive index. This change is particularly significant for gases with high refractive indices, such as acrolein (1.416) [54], triethylamine (1.404) [55], and undecane (1.404) [56]. Additionally, highly polar gases (e.g., methanol, butanol, acetic acid, and acetaldehyde) exhibit attraction to polar groups on the CDs [57], leading to increased adsorption, which promotes changes in the effective refractive index of the PC system.

Illustrations in Fig. 5b, c show the functional group conferred by CDs on the HMSS. The N–O part of the pyridine N-oxide functional group can serve as an effective electron donor, while chloroform is electron-deficient and can act as an electron acceptor [27]. The results demonstrate the critical role of the pyridine N-oxide moiety in chloroform gas detection. Upon comparison, it was found that the HMSS@CDs-PCs sensor’s sensitivity to chloroform vapor was much higher than that of the other seven analyte vapors, indicating that the sensor has good selectivity. Compared to HMSS-PCs, the selectivity of the HMSS@CDs-PCs sensor is enhanced nearly fivefold after CDs functionalization (Fig. 5b, c). The reason for the high selectivity for chloroform vapor is that the CDs loaded in the HMSS material contain pyridinic N-oxide groups. The binding effect of the pyridinic N-oxide group with chloroform has been confirmed [27].

When exposed to 1000 ppm chloroform vapor, the redshift of the reflection spectrum of HMSS@CDs-PCs is 83 nm, which is far higher than the 4 nm observed for SiO_2_-PCs (Fig. 5d). Under relatively constant ambient temperature and relative humidity, the reflection spectrum of HMSS@CDs-PCs undergoes a linear redshift as the chloroform vapor concentration increases from 0 to 500 ppm (Fig. 5e). HMSS@CDs-PCs creates a functionalized hollow mesoporous structure, resulting in an extraordinarily sensitive response to chloroform vapor. The sensitivity of the HMSS@CDs-PCs sensor is 0.79 nm ppm^−1^ (Fig. 5f) with an impressively low LOD of 3.22 ppm, which is the best reported values in fast-response chloroform vapor sensor without multi-signal assistance (Table 1). Compared to traditional PC gas sensors (such as SiO_2_-PCs), the sensitivity of HMSS@CDs-PCs is improved by an order of magnitude. The optical response of the prepared HMSS@CDs-PCs sensor to various chloroform concentrations was tested. The sensitivity of HMSS@CDs-PCs was significantly greater than that of SiO_2_-PCs and MSS-PCs. When the structure of the material changes from solid spheres to hollow mesoporous spheres, the porosity of the system increases, and the effective refractive index also increases, which ultimately results in an enhancement in sensitivity.

**Table 1:** Comparison of the chloroform vapor sensor to previous work

Sensor type	Material	Output signal	S_*n*_ (ppm^−1^)	LOD (ppm)	*t*_*res*_ (s)	*t*_*rec*_ (s)	Refs.
Electric	ZnO/CuO/Al_2_O_3_	$R/R_{0}$	8.5	1	15	> 500	[59]
Electric	GFET	$\Delta R/R_{0}$	0.03	100	–	–	[60]
Electric	Cu/PANi	$\Delta R/R_{0}$	0.02	< 100	–	–	[61]
Optical	PMMA-LSPR	$\Delta E^{*}$	4.76 × 10^–5^	21	30	330	[62]
Electric	PPy/PU	$\Delta R/R_{0}$	0.36	150	36	–	[63]
Electric	FY2 LB	$\Delta f$	5.32 × 10^−4^	1.12 × 10^4^	4	3	[64]
Optical	phosphonated calix	$\Delta I/I_{0}$	0.54	1.23 × 10^4^	120	–	[65]
Optical	PDMS/PSPI	$\Delta W$	3.3 × 10^−4^	–	–	–	[13]
Optical	HMSS@CDs	$\Delta W$	0.79	3.22	7.5	9	This work

S_n_: Sensitivity; LOD: Limit of detection; t_res_: Response time; t_rec_: Recovery time; ${\text{R}}$: Resistance; ${\text{E}}^{*}$: Maximum extinction efficiency; ${\text{f}}:$ Resonance frequency; ${\text{I}}$: Intensity of reflected light; ${\text{W}}:$ Reflection peak wavelength.

The dynamic reflection spectrum response to chloroform vapor was examined in both positive and negative responses. By closing or opening the sealed chamber’s ventilation valve, the sensor’s positive response time is 7.5 s and the negative response time is 9 s (Fig. 5g). When the atmospheric environment in the sealed chamber changes between air containing saturated chloroform vapor and clean air, the response of the reflectance spectrum is rapid and reversible. After nine cycles, the spectral changes of the HMSS@CDs-PCs sensor remain stable (Fig. S4). The peak area of the reflectance spectrum can be used to evaluate the PC effect. The sharper the peak shape, the better the PC effect [58]. In complex environments, the degree of peak shape maintenance is one of the evaluation indicators of sensor stability. The stability of the PC effect of HMSS@CDs-PCs under different standard relative humidity conditions created by saturated salt solution and temperature conditions was tested. The PC effect of HMSS@CDs-PCs is minimally affected by changes in relative humidity and temperature, indicating that the material itself has good moisture resistance and temperature stability (Fig. 5h, i). As the periodicity of the material increases, the PC effect also increases. However, the absolute value of the peak area at different temperatures is not very different (less than 10%). To investigate the reproducibility of the preparation method and sensing performance, the reflectance spectrum redshift of different batches of HMSS@CDs-PC sensor was studied (Fig. S10). The results showed that the reflection peak positions of materials synthesized from different batches were very similar. Furthermore, under 100 ppm chloroform vapor, the redshift of the reflection peaks across different batches was also highly consistent, with an error of less than 3%. These findings indicate that the PC gas sensor preparation method used in this study exhibits excellent reproducibility.

### Sensing Enhancement Mechanisms of PC Gas Sensor

**Fig. 6:**
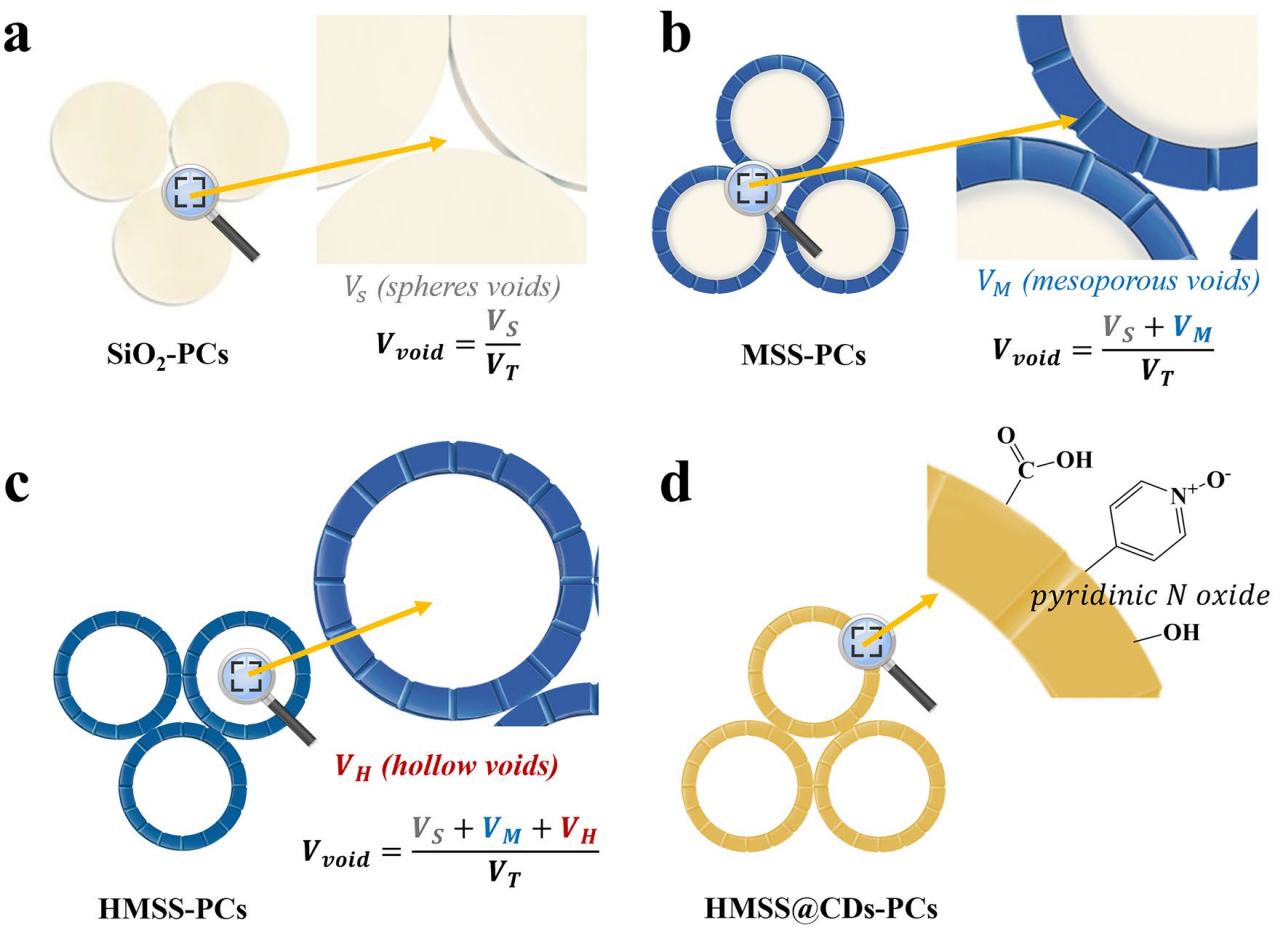
Sensing enhancement mechanisms of PC gas sensor. **a** The units of the PC sensor are composed of solid SiO_2_. **b** The units of the PC sensor are composed of MSS. **c** The units of the PC sensor are composed of HMSS. **d** The units of the PC sensor are composed of HMSS@CDs. The PC gas sensor composed of HMSS (Hollow Mesoporous Silica Spheres) exhibits higher sensitivity. Introducing CDs into the PC system can effectively enhance the selectivity of the PC gas sensor for chloroform

The presence of mesopores is confirmed by the characteristics of typical mesoporous structures, namely type IV curves that appear at P/P_0_ = 0.8–1.0 (Fig. 6a) [66]. The textural properties of HMSS-s and HMSS@CDs-s, two types of hollow microspheres, were further verified by N_2_ adsorption–desorption measurements. The MSS-s possess a narrow pore size distribution centered at about 4 nm. Compared to MSS-s, HMSS-s and HMSS@CDs-s, due to their hollow structure, also exhibit pore size distributions centered at 6 and 11 nm (Fig. 6b). With the addition of mesoporous and hollow structures, the specific surface area, pore size, and pore volume increase (Table S1). After loading CDs, there is a certain degree of reduction in pore size and volume, but the reduction is not significant. This demonstrates that CDs are only loaded into mesopores and do not enter the cavities of HMSS. This is because the material, in order to retain active functional groups, has not undergone high-temperature calcination, resulting in irregular pore structures. These irregular pores prevent CDs from entering the cavities, ensuring high specific surface area and porosity of the material [67]. This also corresponds to the conclusion drawn from the N_2_ adsorption–desorption isotherm analysis.

According to the simplified Bragg’s Law, when light illuminates a PC, the periodic modulation of the crystal generates a band structure, resulting in a photonic band gap. As a consequence of this band gap, light waves of this energy cannot propagate through the crystal. The simplified Bragg’s Law can be expressed by:

3 $$\begin{array}{*{20}c} {\lambda = \sqrt{\frac{8}{3}} D\sqrt {n_{{{\text{eff}}}}^{2} - \sin^{2} \theta } } \\ \end{array}$$

where $\lambda$ is diffraction peak wavelength, $D$ is particle diameter, and $\theta$ is light incidence angle. Here $n_{{{\text{eff}}}}$ is effective refractive index, which is expressed as:

4 $$\begin{array}{*{20}c} {n_{{{\text{eff}}}}^{2} = n_{{\text{p}}}^{2} V_{{\text{p}}} + n_{{{\text{void}}}}^{2} V_{{{\text{void}}}} } \\ \end{array}$$

where $n_{{\text{p}}}$ is refractive indices of particles, $n_{{{\text{void}}}}$ is refractive indices of void, $V_{{\text{p}}}$ is volume fractions of particles and $V_{{{\text{void}}}}$ is volume fraction of void, which is expressed as:

5 $$\begin{array}{*{20}c} {V_{{\text{p}}} + V_{{{\text{void}}}} = 1} \\ \end{array}$$

When the PC sensor detects the gas, the variation in the reflection spectrum using the following equation:

6 $$\begin{array}{*{20}c} {\Delta \lambda = \lambda_{Gas} - \lambda_{Air} = \sqrt{\frac{8}{3}} D\left[ {\sqrt {n_{p}^{2} \left( {1 - V_{{{\text{void}}}} } \right) + n_{{{\text{Gas}}}}^{2} V_{{{\text{void}}}} - \sin^{2} \theta } - \sqrt {n_{p}^{2} \left( {1 - V_{{{\text{void}}}} } \right) + n_{{{\text{Air}}}}^{2} V_{{{\text{void}}}} - \sin^{2} \theta } } \right]\# } \\ \end{array}$$

where ${\Delta }\lambda$ is reflectance wavelength change, $n_{{{\text{Gas}}}}$ is refractive index of gas, $n_{{{\text{Air}}}}$ is refractive index of air. Equation 6 can be viewed as a function of $V_{{{\text{void}}}}$, which is expressed as:

7 $$\begin{array}{*{20}c} {\Delta \lambda = f\left( {V_{{{\text{void}}}} } \right)} \\ \end{array}$$

According to the derivation (Fig. S5), $f\left( {V_{{{\text{void}}}} } \right)$ is an increasing function. This means that according to the definition of sensitivity, the sensitivity of the PC gas sensor increases with the increase in porosity, which is expressed as:

8 $$\begin{array}{*{20}c} {{\text{S}}_{n} \propto V_{{{\text{void}}}} } \\ \end{array}$$

According to Eq. 8, it can be concluded that the sensitivity of PC gas sensors increases with the increase in the void volume fraction ($V_{{{\text{void}}}}$). In other words, increasing the $V_{{{\text{void}}}}$ is an effective method to enhance the sensitivity of PC gas sensors.

In this study, the incorporation of mesoporous and hollow structures effectively increases the $V_{{{\text{void}}}}$ in the PC system. The underlying reason is as follows: the structural units are arranged in a face-centered cubic (FCC) configuration. When the units are composed of solid SiO_2_, the $V_{{{\text{void}}}}$ is equal to the ratio of the void volume between the spheres ($v_{{\text{s}}}$) to the total volume ($v_{{\text{T}}}$), which can be expressed as:

9 $$\begin{array}{*{20}c} {V_{{{\text{void}}}} \left( {{\text{SiO}}_{2} } \right) = \frac{{v_{{\text{s}}} }}{{v_{{\text{T}}} }}} \\ \end{array}$$

where $V_{{{\text{void}}}}$ is void volume fraction, $v_{{\text{s}}}$ is the void volume between the spheres, $v_{{\text{T}}}$ is total volume. When the units are MSS, the $V_{{{\text{void}}}}$ is the ratio of the $v_{{\text{s}}}$ plus the void volume in the mesoporous ($v_{{\text{m}}}$) to the $v_{{\text{T}}}$. This can be expressed as:

10 $$\begin{array}{*{20}c} {V_{{{\text{void}}}} \left( {{\text{MSS}}} \right) = \frac{{v_{{\text{s}}} + v_{{\text{m}}} }}{{v_{{\text{T}}} }}} \\ \end{array}$$

where $v_{{\text{m}}}$ is void volume in the mesoporous. When the primitive is hollow mesoporous HMSS, the void volume fraction is equal to the ratio of VS plus VM plus the void volume in the hollow cavity ($v_{{\text{H}}}$) to the total volume. This can be expressed as:

11 $$\begin{array}{*{20}c} {V_{{{\text{void}}}} \left( {{\text{HMSS}}} \right) = \frac{{v_{{\text{s}}} + v_{{\text{m}}} + v_{{\text{H}}} }}{{v_{{\text{T}}} }}} \\ \end{array}$$

where $v_{{\text{H}}}$ is void volume in the hollow cavity. It can be observed that HMSS possess a larger void volume fraction, i.e.,

12 $$\begin{array}{*{20}c} {V_{{{\text{void}}}} \left( {{\text{HMSS}}} \right) > V_{{{\text{void}}}} \left( {{\text{MSS}}} \right) > V_{{{\text{void}}}} \left( {{\text{SiO}}_{2} } \right)} \\ \end{array}$$

According to Eq. 8, it can be concluded that the PC gas sensor composed of HMSS (hollow mesoporous silica spheres) exhibits higher sensitivity.

Pyridine N-oxide group-functionalized CDs exhibit a specific affinity for chloroform, as confirmed by previous studies [27]. In simple terms, the N–O portion of the pyridine N-oxide functional group can serve as an effective electron donor, while chloroform acts as an electron acceptor. Introducing these CDs into the PC system can effectively enhance the selectivity of the PC gas sensor for chloroform. Additionally, the mesoporous and hollow structures do not appear to introduce any changes to the functional groups of CDs and therefore have no effect on the selectivity for chloroform.

## Conclusions

In summary, we have developed a CPC chloroform vapor sensor self-assembled by CDs-modified hollow mesoporous silica spheres. The sensor operates based on the bandgap (stopband) shift as a function of the effective refractive index change. When the analyte gas enters the CPC through mesopores, it alters the effective refractive index, causing changes in the reflectance spectrum. The optical response of the HMSS@CDs-PCs sensor to different concentrations of chloroform vapor was measured using a reflectance spectrum test platform equipped with a fiber optic spectrometer, achieving a sensitivity of 0.79 nm ppm^−1^ and LOD of 3.32 ppm. In addition, a fast, stable, and reversible optical response of the HMSS@CDs-PCs sensor was observed in the positive and negative test cycles of chloroform sensing. The positive response time is 8 s, and the negative response time is 6 s. Lastly, stability and repeatability tests showed that the sensor maintained stable performance in humidity levels between 20% and 85%RH and temperatures ranging from 25 to 55 °C. The sensor developed here also demonstrated strong selectivity for chloroform vapor even in the presence of six vapors with similar properties and two representative acidic and basic gases.

Our study demonstrates that monodispersed HMSS are excellent building blocks for CPC-based optical sensors, because they form interconnected hierarchically ordered macro-mesoporous structures without the need of structure inversion. CDs are shown to be an excellent ‘nanoreceptors’ to couple into the HMSS platform owing to the size match of the CDs and the mesopores. The proposed design concept of 'Nanoreceptors' demonstrates significant research potential in the field of gas sensors. By modulating the structure and functionality of molecular receptors, it enables customized designs for different gases and facilitates the development of sensor arrays capable of accurately distinguishing multiple gases in complex environments, thereby greatly enhancing sensor selectivity and sensitivity. Moreover, this concept is expected to drive the advancement of gas sensors toward intelligent and integrated systems, offering innovative solutions for applications in environmental monitoring, medical diagnostics, and industrial safety.

## Supplementary Information

Below is the link to the electronic supplementary material.

Supplementary file1 (DOCX 10124 KB)

Supplementary file2 (MP4 830 KB)
