# Supplementary material for: Carbon Dots-Modified Hollow Mesoporous Photonic Crystal Materials for Sensitivity- and Selectivity-Enhanced Sensing of Chloroform Vapor
Source: Nanomicro Lett. 2024 Dec 26;17:96. doi: 10.1007/s40820-024-01598-9 (PMC11671676; doi:10.1007/s40820-024-01598-9)
Supplement: Supplementary file 1 — Supplementary file1 (DOCX 10124 KB) [file 40820_2024_1598_MOESM1_ESM.docx]

Supporting Information for

**Carbon Dots-Modified Hollow Mesoporous Photonic Crystal Materials for Sensitivity and Selectivity Enhanced Sensing of Chloroform Vapor**

Junchen Liu^1,2,3^, Ji Liu^1^, Zhipeng Li^1^, Liupeng Zhao^1,^ *, Tianshuang Wang^1^, Xu Yan^1^, Fangmeng Liu^1^, Xiaomin Li^2,^ *, Qin Li^3,^ *, Peng Sun^1,^ *, Geyu Lu^1^, Dongyuan Zhao^2^

^1^ State Laboratory on Integrated Optoelectronics, College of Electronic Science and Engineering, Jilin University, Changchun 130012, P. R. China

^2^ Department of Chemistry and Laboratory of Advanced Materials, Shanghai Key Laboratory of Molecular Catalysis and Innovative Materials, State Key Laboratory of Molecular Engineering of Polymers, Collaborative Innovation Center of Chemistry for Energy Materials (2011-iChEM), College of Chemistry and Materials, Fudan University, Shanghai, P. R. China

^3^ School of Engineering and Built Environment, Griffith University, Nathan, QLD 4111, Australia

*Corresponding authors. E-mail: [zhaoliupeng@jlu.edu.cn](mailto:zhaoliupeng@jlu.edu.cn) (Liupeng Zhao); [lixm@fudan.edu.cn](mailto:lixm@fudan.edu.cn) (Xiaomin Li); [qin.li@griffith.edu.au](mailto:qin.li@griffith.edu.au) (Qin Li); [pengsun@jlu.edu.cn](mailto:pengsun@jlu.edu.cn) (Peng Sun)

**S1 Schematic diagram of gas sensing test platform of self-made photonic crystal gas sensor**

The photonic crystal gas sensor converts the information about gas type and concentration into the information from the reflection spectrum redshift [S1]. Therefore, effective characterization of the reflection spectrum of photonic crystals is key to gas sensitivity [S2]. A sealed test chamber was developed. The test chamber is composed of an incident fiber, exit fiber, integrating sphere, humidity detector, and temperature detector. A halogen light source and optical fiber spectrometer are connected to the outside of the test chamber. The target gas was injected into the closed chamber, and the change in the reflectance spectrum of the photonic crystal film was monitored. During baseline testing, dry air was selected as the gas medium. Some VOC vapor was introduced by injecting a small amount of VOC liquid into a closed container and allowing it to evaporate. The schematic diagram of the gas sensing test platform of the self-made photonic crystal gas sensor is displayed in **Fig. S1**.

**
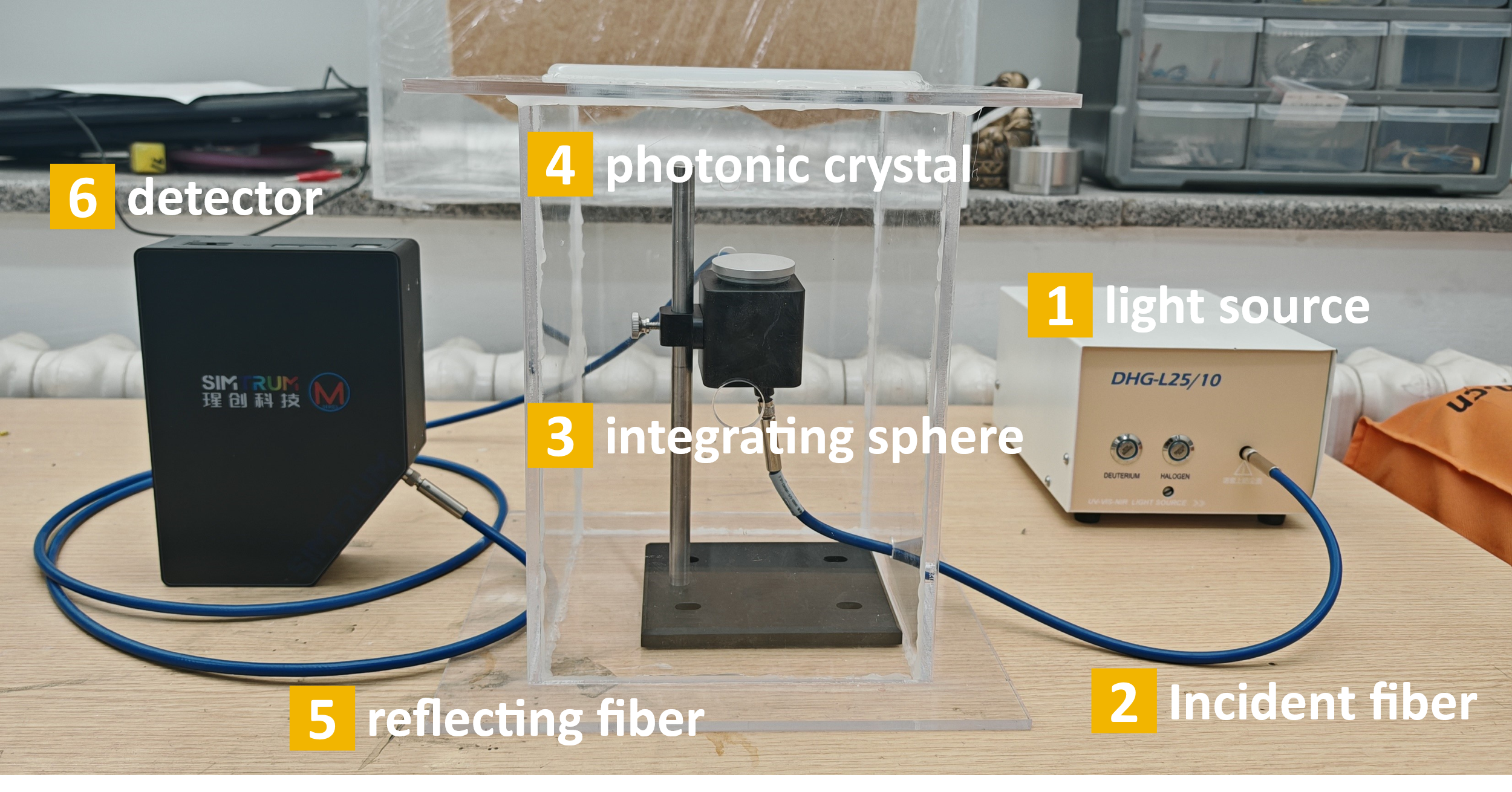
**

**Fig. S1** schematic diagram of gas sensing test platform of self-made photonic crystal gas sensor

**S2 Calculation of noise value**

S2 Calculation of noise value. Under normal temperature and pressure conditions, the photonic crystal gas sensor is stabilized with clean air. The peak position (nm) of the standard reflection whiteboard's reflection spectrum is recorded every 2 minutes. This process is continued until 30 data points are collected. The noise value is then calculated using the following Equation S1 [S3].

$$\begin{aligned} \sigma=\sqrt{\frac{\sum_{=1}^{n} {(\lambda_{i}-\lambda)}^{2}}{n-1}}\#\left( S1 \right) \end{aligned}$$

where $\sigma$ is noise value, $\lambda_{i}$ is the ith measurement value, $\lambda$ is the average measurement value, $n$ is total number of measurement data, $i$ is measurement data serial number. The calculated $\sigma$ is 0.85. The peak positions of the standard reflection board's reflection spectrum are recorded and displayed in **Fig. S2.**


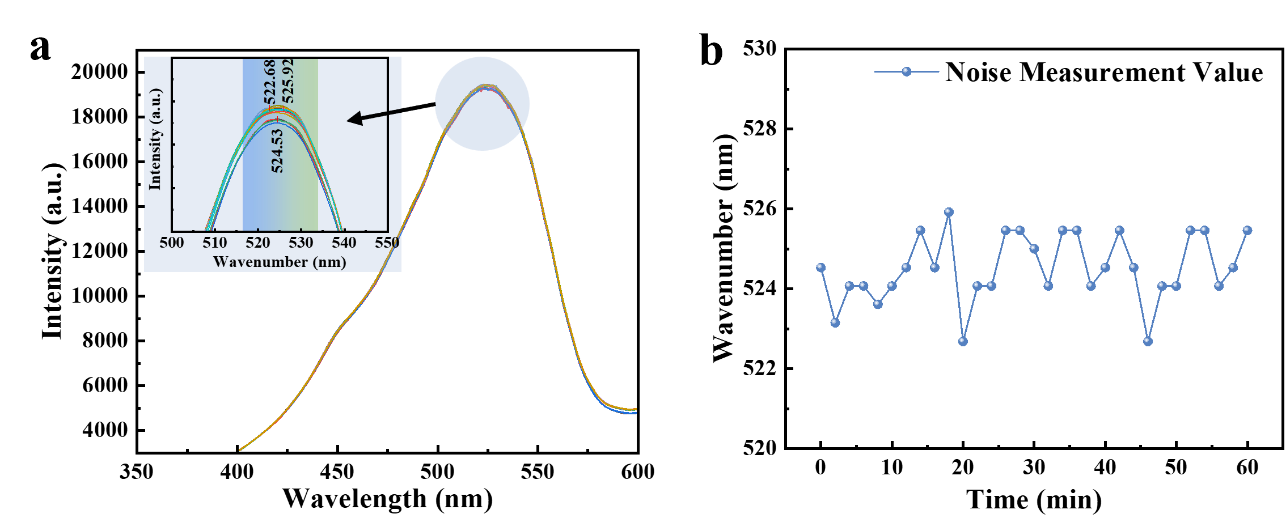


**Fig. S2** **Calculation of noise value**. **a** Reflection spectrum recorded every two minutes. **b** Peak positions of the standard reflection board's reflection spectrum recorded every 2 minutes

**S3 Fluorescence effects of HMSS@CDs-aq and HMSS@CDs-PC film**

Under a 365 nm UV lamp, HMSS@CDs composite still fluoresce in ethanol solution or after self-assembly into a film. This indicates that there is no aggregation-induced quenching effect in CDs. The special structure of HMSS makes CDs disperse. The optical photograph of fluorescence effects of HMSS@CDs-aq and HMSS@CDs-PC film is displayed in **Fig. S3.**


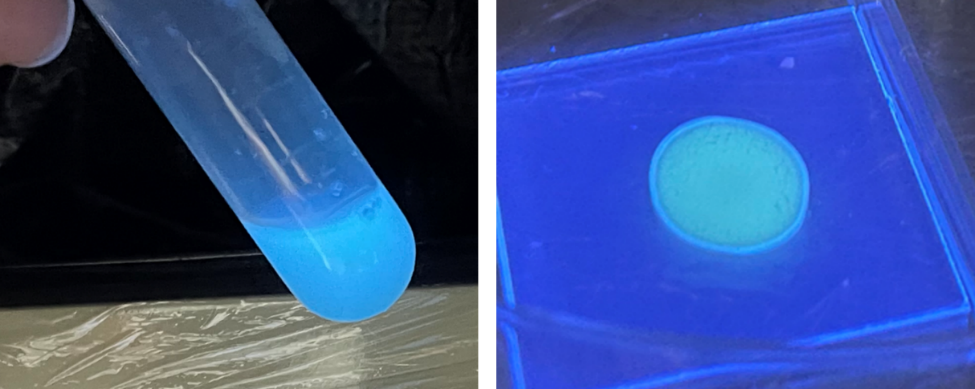


**Fig. S3** Fluorescence effects of HMSS@CDs-aq and HMSS@CDs-PC film

**S4 Stability test of HMSS@CDs-PCs**

Clean air and chloroform vapor were successively passed into the test chamber nine times to record the red shift of the reflection spectrum. It can be observed that the reflected spectrum always returns to its original position when the clean air environment is restored in the test chamber. The cyclic stability test of HMSS@CDs-PCs is displayed in **Fig. S4.**


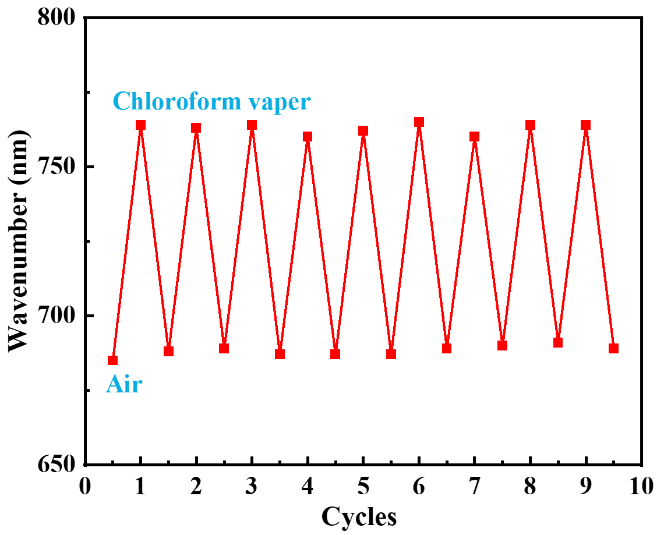


**Fig. S4** Chloroform vaper sensing cyclic stability test of HMSS@CDs-PCs

**S5 Relationship between sensitivity and porosity of a photonic crystal gas sensor**

When the PC sensor detects the gas, the variation of the reflection spectrum using the following equation:

$$\begin{aligned} \Delta\lambda=f\left( x \right)=\sqrt{\frac{8}{3}}d\left[ \sqrt{n_{p}^{2}\left( 1-x \right){{+n}_{\mathrm{Gas}}}^{2}x-\sin^{2} \theta}-\sqrt{n_{p}^{2}\left( 1-x \right){{+n}_{\mathrm{Air}}}^{2}x-\sin^{2} \theta} \right]\#(S2) \end{aligned}$$

Reduce to:

$$\begin{aligned} \left\{ \begin{aligned} \Delta\lambda=f\left( x \right)=A\left[ \sqrt{Bx+C}-\sqrt{Dx+C} \right] \\ A=\sqrt{\frac{8}{3}}d \\ B={{n_{\mathrm{Gas}}}^{2}-n}_{p}^{2} \\ C=n_{p}^{2}-\sin^{2} \theta\\ D={n_{\mathrm{Air}}}^{2}{-n}_{p}^{2} \end{aligned} \right.\#(S3) \end{aligned}$$

The domain of this function is:

$$\begin{aligned} 0<x<1\#\left( S4 \right) \end{aligned}$$

The image of the Function S3 under the domain was shown in **Fig. S5.** According to the function image, it can be seen that this function is an increasing function. This means that according to the definition of sensitivity, the sensitivity of the photonic crystal gas sensor increases with the increase of porosity.


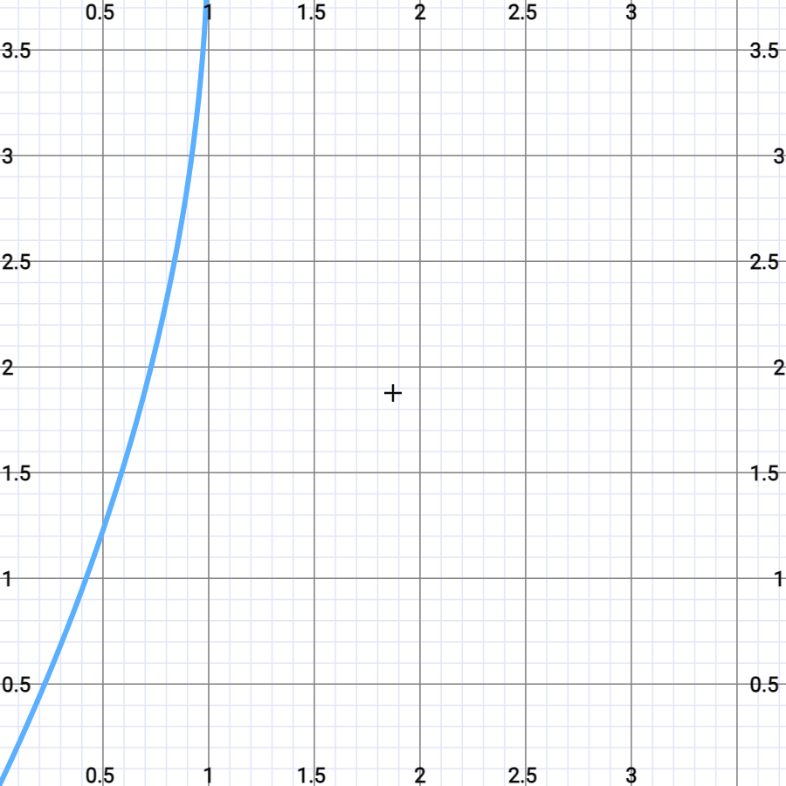


**Fig. S5** Image of this function under the domain

**S6 High-resolution O 1s spectrum of HMSS-s**

To visually compare the modification of the material by carbon dots, the XPS spectra of O 1s for HMSS-s were displayed in **Fig. S6**.


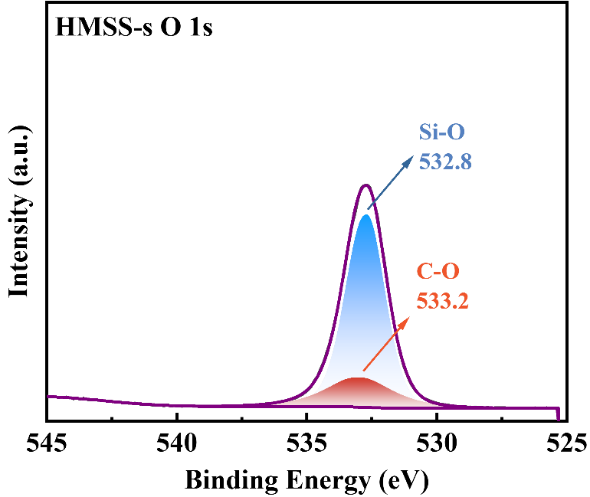


**Fig. S6** High-resolution O 1s spectrum of HMSS-s

**S7 Sensing capability test with high concentration**

A sensing capability test under 500 ppm vapor concentration of HMSS@CDs-PC was displayed in **Fig. S7**.


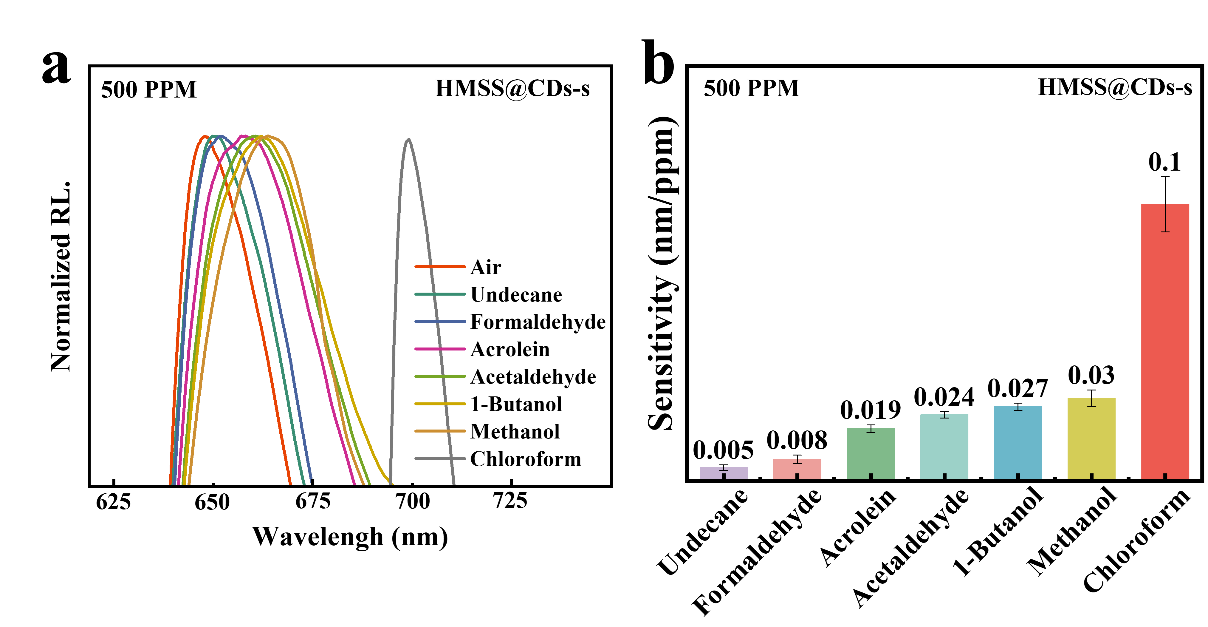


**Fig. S7 Sensing capability test with high concentration.** **a** Reflection spectrum of HMSS@CDs-PCs sensor under different kinds of 500 ppm VOC atmospheres. **b** Selectivity HMSS@CDs-PCs sensor under different kinds of 500 ppm VOC atmospheres

**S8 Optical microscope image of HMSS@CDs-PC**

The defects in the films were observed using an optical microscope in Fig. S8


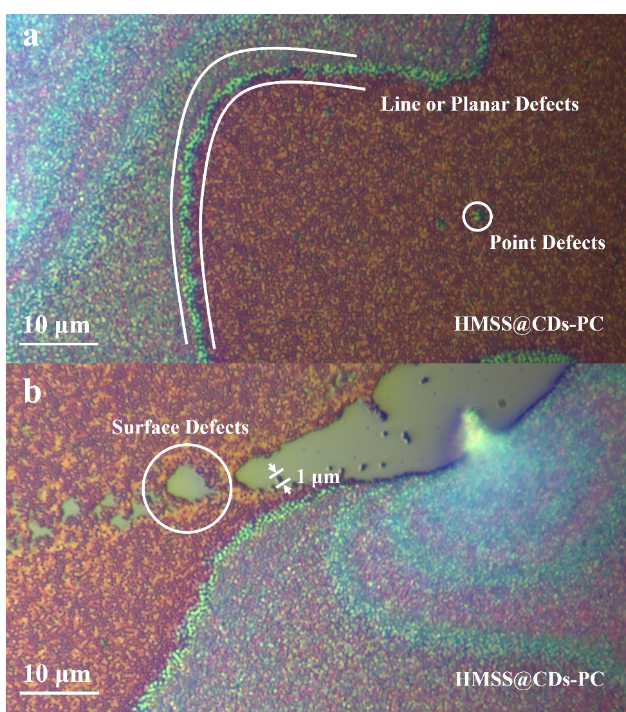


**Fig. S8** Optical microscope image of HMSS@CDs-PC

**S9 Selectivity test of HMSS@CDs-PC**

Selectivity test of HMSS@CDs-PC under 100 ppm acetic acid and triethylamine was displayed in **Fig. S9.**


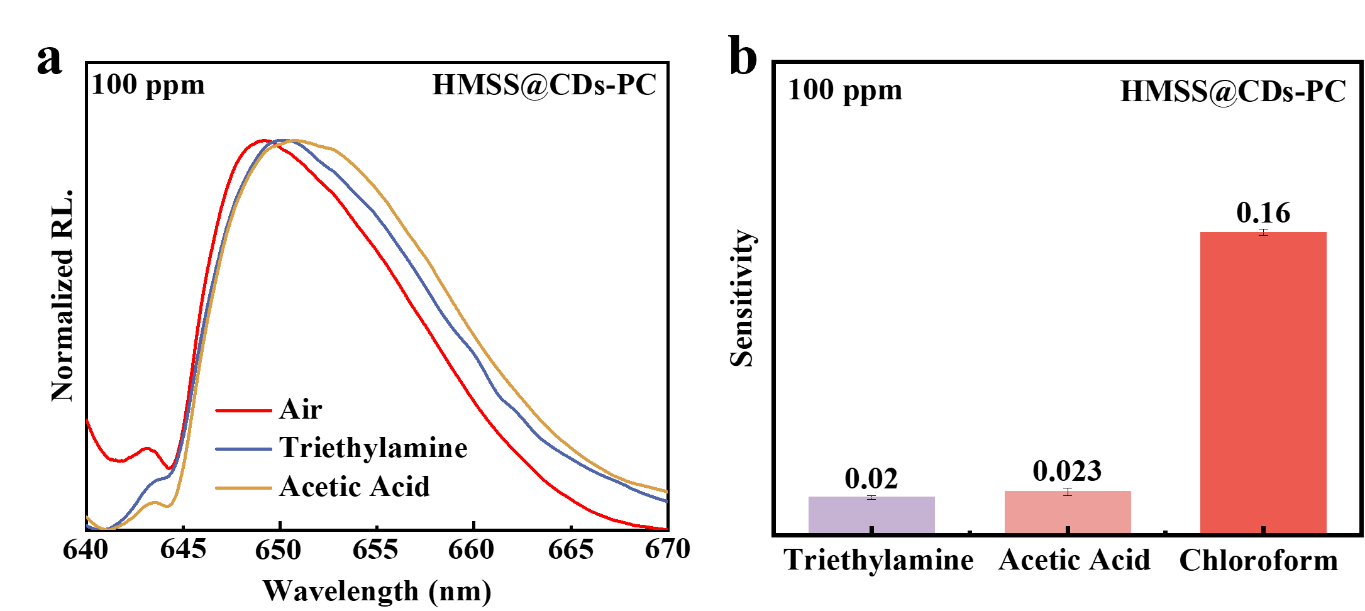


**Fig. S9** **Selectivity test of HMSS@CDs-PC.** **a**. Red-shift of reflection spectrum under 100 ppm triethylamine and Acetic Acid. **b**. Sensitivity of HMSS -PCs sensor under 100 ppm triethylamine and Acetic Acid

**S10 Reproducibility of HMSS@CDs-PC**

Reproducibility of HMSS@CDs-PC by studying the reflectance spectra and the redshift of spectra for materials synthesized in different batches was displayed in **Fig. S11**.


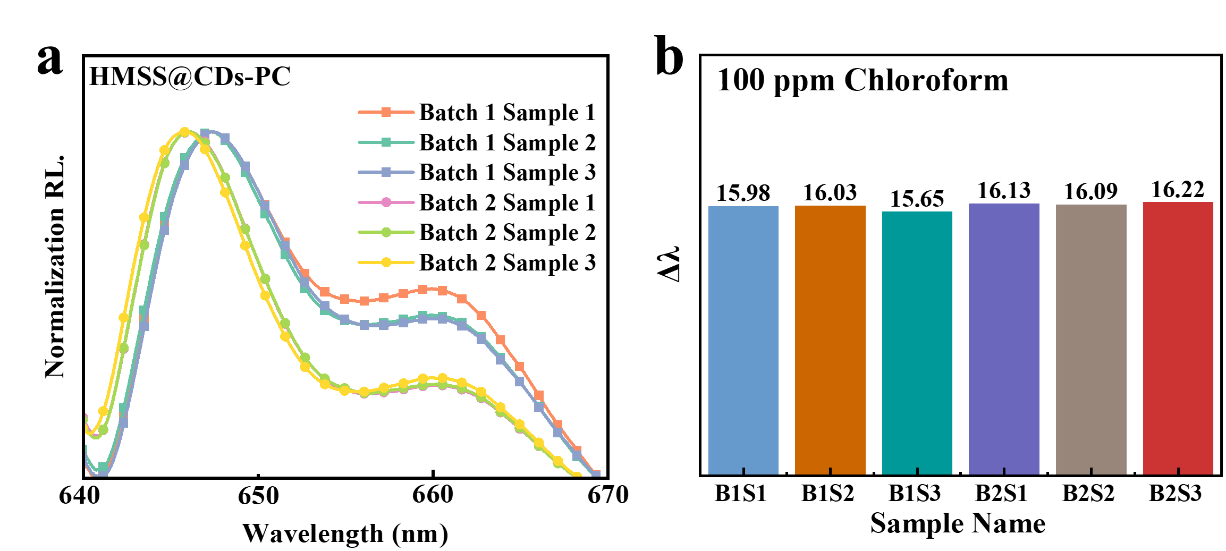


**Fig. S10 Reproducibility of HMSS@CDs-PC.** **a** Reflectance spectra for materials synthesized in different batches. **b** Redshift of spectra for materials synthesized in different batches

**S11 Nitrogen adsorption-desorption isotherms**

Nitrogen adsorption-desorption isotherms of SiO_2_-s, MSS-s, HMSS-s, and HMSS@CDs-s were displayed in **Fig. S11**.


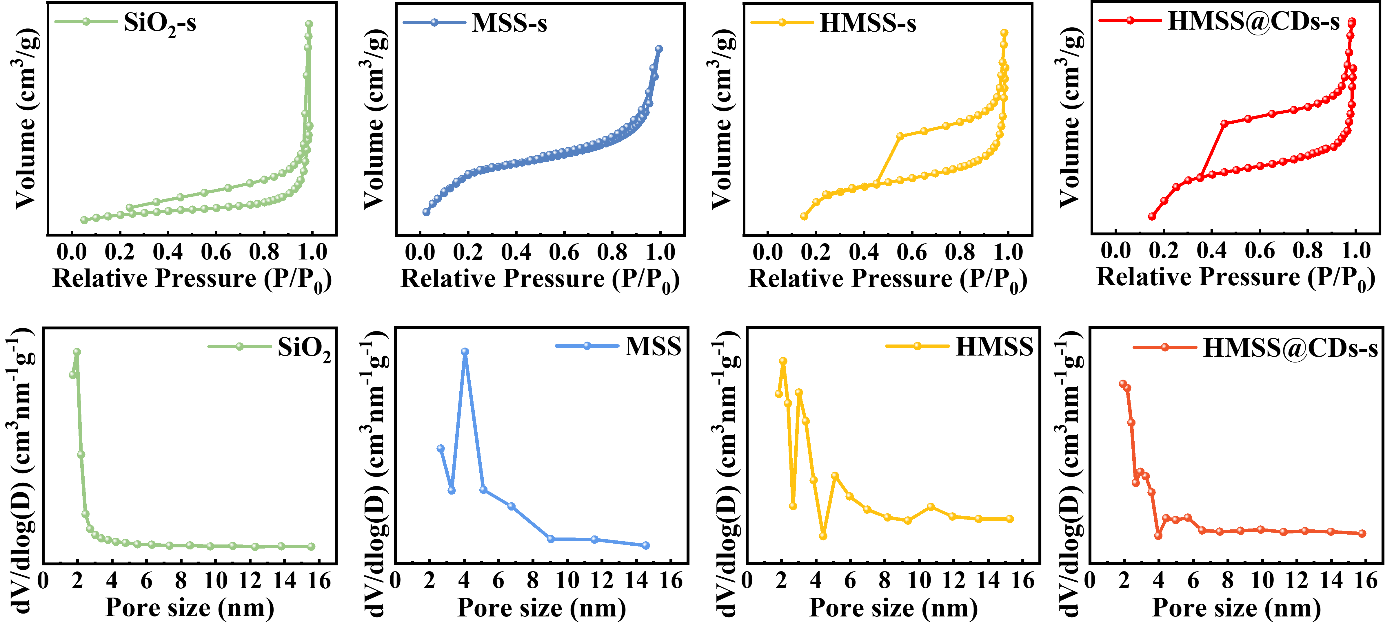


**Fig. S11 N_2_ adsorption-desorption isotherms of SiO_2_-s, MSS-s, HMSS-s, and HMSS@CDs-s.** N_2_ adsorption-desorption isotherms of SiO_2_-s, MSS-s, HMSS-s, and HMSS@CDs-s. The isotherm of SiO_2_-s is classified as a type II isotherm, indicating its non-porous structure. MSS-s exhibits a type IV curve at P/P0=0.8~1.0, indicating the presence of mesopores. The hysteresis loop observed for HMSS-s and HMSS@CDs-s at P/P0=0.4~1.0 is caused by the hollow structure. The illustration shows the tendency of the volume fraction of voids to increase as the structure changes. BJH desorption pore distribution report of SiO_2_-s, MSS-s, HMSS-s, and HMSS@CDs-s. The pore size distribution of MSS-s shows a narrow peak at 4-10 nm, indicating a high uniformity of the mesopore sizes

**S12 Small Angle XRD pattern of HMSS-s and HMSS@CDs-s**

Small Angle XRD pattern of HMSS-s and HMSS@CDs-s was displayed in **Fig. S12**


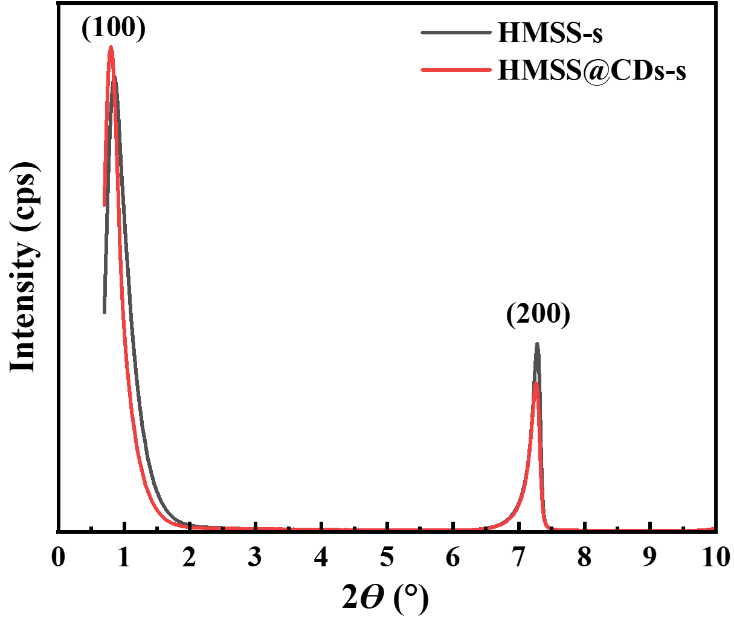


**Fig. S12** Small Angle XRD pattern of HMSS-s and HMSS@CDs-s

**S13 BET Surface Area and Desorption average pore diameter**

The specific surface area and pore size of the sample were analyzed using QUADRASORB evo, and the results are shown in **Table S1**.

| **Table S1** BET Surface Area and Desorption average pore diameter | | |  |
| --- | --- | --- | --- |
|  | ***BET Surface Area (m^2^/g)*** | ***Desorption average pore diameter (nm)*** | ***Total pore volume (cm^3^/g)*** |
| SiO_2_-s | 12.2417 | 2.4673 | 0.32757 |
| MSS-s | 273.0968 | 5.6312 | 0.94089 |
| HMSS-s | 500.1660 | 11.8879 | 1.57514 |
| HMSS@CDs-s | 493.6586 | 7.3910 | 1.16209 |

**Supplementary References**

1. Z.A. Zaky, A.M. Ahmed, A.S. Shalaby, A.H. Aly, Refractive index gas sensor based on the tamm state in a one-dimensional photonic crystal: Theoretical optimisation. Sci. Rep. **10**(1), 9736 (2020). <https://doi.org/10.1038/s41598-020-66427-6>
2. R.A. Potyrailo, H. Ghiradella, A. Vertiatchikh, K. Dovidenko, J.R. Cournoyer et al., Morpho butterfly wing scales demonstrate highly selective vapour response. Nat. Photonics **1**(2), 123-128 (2007). <https://doi.org/10.1038/nphoton.2007.2>
3. M. Vlk, A. Datta, S. Alberti, H.D. Yallew, V. Mittal et al., Extraordinary evanescent field confinement waveguide sensor for mid-infrared trace gas spectroscopy. Light: Sci. Appl. **10**(1), 26 (2021). <https://doi.org/10.1038/s41377-021-00470-4>
